# Phycocyanobilin: A potential bioactive compound from *Arthrospira platensis* targeting LYN protein associated with systemic lupus erythematosus

**DOI:** 10.1371/journal.pone.0357093

**Published:** 2026-08-28

**Authors:** Amnart Chaiprasert, Ping Han, Teeraphan Laomettachit, Marasri Ruengjitchatchawalya

**Affiliations:** 1 Biotechnology Program, School of Bioresources and Technology (SBT), King Mongkut's University of Technology Thonburi (KMUTT), Bangkok, Thailand; 2 Department of Medicine, Phramongkutklao College of Medicine, Bangkok, Thailand; 3 Bioinformatics and Systems Biology Program, SBT, KMUTT, Bangkok, Thailand; 4 Bioinformatics and Systems Biology Program, School of Information Technology, KMUTT, Bangkok, Thailand; 5 Algal Biotechnology Research Group, Pilot Plant Development and Training Institute (PDTI), KMUTT, Bangkok, Thailand; Mansoura University, EGYPT

## Abstract

Phycocyanobilin, a bioactive compound derived from *Arthrospira platensis* C1, was investigated for its potential role in systemic lupus erythematosus (SLE) based on its structural similarity to bilirubin, with a Tanimoto score of 93%. Molecular docking revealed favorable binding affinities between phycocyanobilin and several protein targets, including EGFR, FYN, HLA-B, LCK, LYN, and TP53. Target prediction further identified LYN kinase as a key candidate. Molecular dynamics simulations demonstrated stable binding of the phycocyanobilin–LYN complex, with interaction profiles comparable to those of the native ligand, staurosporine. Binding free energy and residue-level analyses supported strong and stable interactions, highlighting key contributions to complex stability. Overall, these findings provide mechanistic insight into the interaction between phycocyanobilin and LYN, suggesting that this compound may modulate LYN-associated signaling pathways and warrants further investigation in the context of SLE.

## Introduction

Systemic lupus erythematosus (SLE) is an autoimmune disease with a broad spectrum of clinical manifestations and an unclear etiology. The disease affects multiple organ systems and is associated with increased morbidity and mortality [1,2]. Although immunosuppressive drugs can induce disease remission, relapses may still occur and are often unpredictable. Various factors, including sunlight exposure and stress, can trigger disease flares. Serum bilirubin, a product of heme degradation traditionally considered a marker of liver disease, has been shown to correlate negatively with disease activity in patients with SLE [3]. Reduced serum bilirubin levels in SLE patients may be associated with inflammatory processes and lupus-related kidney involvement [3–5]. Furthermore, patients with inactive SLE exhibit higher bilirubin levels than those with active disease, and patients without lupus nephritis have higher serum bilirubin levels than those with nephritis [6]. In addition, bilirubin has emerged as a potent signaling molecule with strong antioxidant properties. It exerts broad inhibitory effects on multiple components of the immune system, contributing to protection against autoimmune and inflammatory diseases [7].

Spirulina (*Arthrospira* or *Limnospira platensis*), a photosynthetic cyanobacterium, is commonly used as a food and feed supplement due to its rich nutrient content and diverse bioactive compounds. These include the water-soluble phycobiliprotein phycocyanin, which contains phycocyanobilin as a chromophore. Phycocyanobilin belongs to a group of open-chain tetrapyrrole chromophores that bind to proteins via thioester linkages to cysteine residues [8–10]. This microalga exhibits immunomodulatory activity, and its beneficial effects have been investigated in patients with various diseases [11–19]. Our previous study [20] identified bioactive compounds from Spirulina (*A. platensis* C1) associated with immunological responses in SLE using structural similarity, bioassay similarity, disease-drug-compound network analysis, molecular docking, and molecular dynamics (MD) simulations. A high Tanimoto score indicates structural similarity between Spirulina-derived compounds and immunosuppressive agents; although phycocyanobilin was not among the highest-scoring compounds (90–100%), it was identified within a similarity range of ≥60% (see S5 Table in [20]).

In this study, we aimed to investigate the potential of the algal-derived bioactive compounds, particularly phycocyanobilin. Its chemical structure resembles that of bile pigments, including bilirubin, suggesting that it may be associated with SLE or exhibit similar molecular effects.

## Materials and methods

### Structural similarity between bioactive compounds of Spirulina and bilirubin

The structural similarity between bioactive compounds of Spirulina (*A. platensis* C1) and bilirubin was analyzed using the PubChem structural similarity tool based on compound identifiers (CIDs) (https://pubchem.ncbi.nlm.nih.gov/score_matrix/score_matrix.cgi) [21]. A total of 833 bioactive compounds from *A. platensis* C1 were identified from the Kyoto Encyclopedia of Genes and Genomes (KEGG; www.genome.jp/kegg/), the Spirulina Proteome Repository (SpirPro, see S1 Table in [20]), and a literature review [20,22,23]. Structural similarity matching was performed between bilirubin (CID 5280352) and these 833 algal-derived bioactive compounds, including phycocyanobilin [20].

### Molecular docking and molecular dynamics simulations of bioactive compounds and targets

To determine whether phycocyanobilin exhibits molecular signaling effects similar to bilirubin, the binding affinities of these two compounds toward several related receptors were evaluated using molecular docking. This approach aims to identify the most favorable binding modes between ligands and receptors. Molecular docking simulations were performed using MGLTools (http://mgltools.scripps.edu/) with AutoDock. The input files included the 3D structures of the ligands retrieved from PubChem (https://pubchem.ncbi.nlm.nih.gov) and the receptor proteins in PDB format, obtained from the Research Collaboratory for Structural Bioinformatics (RCSB) Protein Data Bank (PDB) (https://www.rcsb.org). Prior to docking, both ligands and receptors were converted from PDB to PDBQT format. Docking simulations were conducted to evaluate the binding of bilirubin and phycocyanobilin to all potential receptors, including EGFR (binding site 1: W2R; binding sites 2 and 3: SO4), FYN, HLA-B, LCK, LYN, and TP53, which were initially identified through our previous report [20]. To ensure comprehensive coverage of potential binding sites, the grid box was configured to encompass the relevant binding domains of each receptor, enabling thorough sampling of potential binding conformations. For the EGFR kinase domain, the grid box specifically included the W2R binding site and two SO4 sites. Detailed grid coordinates and exhaustiveness parameters were defined for each AutoDock run. The reliability of the docking protocol was validated by comparing the predicted binding energies of the studied compounds with those of native ligands from experimentally resolved crystal structures available in the PDB, using the same grid parameters and search settings. The accuracy of the protocol was confirmed by calculating the root-mean-square deviation (RMSD) between the predicted docking poses and the original crystal structures. In addition to docking, NetPredictor (https://github.com/abhik1368/netpredictor), a bioinformatics tool that predicts biological interactions using a network-based approach, was employed to identify potential target receptors of the bioactive compounds. Based on the docking and NetPredictor results, the most promising protein receptor was selected for further investigation using molecular dynamics (MD) simulations. The LYN kinase structure (PDB ID: 3A4O) was prepared by removing all water molecules and heteroatoms to obtain the apo-protein. Protonation states of all ionizable amino acid residues were assigned at pH 7.0 using PROPKA 3.0 to simulate physiological conditions. The structure was then subjected to energy minimization using the sander module in AMBER 16 to resolve steric clashes and optimize geometry. MD simulations were performed for 60 ns using AMBER 16, with the ff14SB force field applied to both the protein and ligand. The system was maintained at a constant temperature of 300 K and a pressure of 1 atm. System setup was carried out using LEaP, and energy minimization was performed using *sander*. Bond and angle constraints were applied using the SHAKE algorithm. System stability was evaluated by calculating the RMSD of the protein backbone, complex, and ligand using the PTRAJ module. The final 10 ns of MD trajectories were extracted for further analysis, including binding free energy calculations, hydrogen bond analysis, and identification of key residues involved in ligand binding. The MM/PBSA and MM/GBSA methods were used to calculate the binding free energy (ΔG_bind) of the simulated complexes. The total binding free energy consisted of entropy, electrostatic energy, van der Waals (vdW) energy, solvation energy, and polar solvation energy. For comparison**,** MD simulations were performed on both the algal-derived compounds in complex with their potential protein receptors and the corresponding native protein–ligand complexes.

## Results

### Molecular docking of bioactive compounds and targets

We first assessed the structural similarity between bioactive compounds of Spirulina (*A. platensis* C1) and bilirubin. The results showed that bilirubin (https://pubchem.ncbi.nlm.nih.gov/compound/5280352#section=2D-Structure) shares a high Tanimoto score of 93% with phycocyanobilin (https://pubchem.ncbi.nlm.nih.gov/compound/137699530#section=2D-Structure), a notable bioactive compound in Spirulina (Figure in S1 Fig). Both bilirubin and phycocyanobilin are involved in the porphyrin biosynthesis pathway (KEGG pathway map00860). Subsequently, molecular docking studies using MGLTools were conducted to evaluate the binding affinities of bilirubin and phycocyanobilin against their potential target receptors, including EGFR, FYN, HLA-B, LCK, LYN, and TP53. Due to the presence of multiple binding sites within the EGFR kinase domain, including one W2R site and two SO_4_ sites, all potential sites were included in the analysis. The grid box was configured to encompass these binding sites, after which bilirubin and phycocyanobilin were docked to each receptor. The AutoDock results for both ligands, bilirubin and phycocyanobilin, against the selected receptors, as obtained using MGLTools, are summarized in Table 1. In addition to AutoDock, we performed molecular docking using FlexX and iGEMDOCK (Figure in S2 Fig). The results from these independent docking methods were consistent with those obtained using AutoDock, with LYN consistently ranking among the highest-scoring targets for phycocyanobilin across all evaluated receptors.

**Table 1:** AutoDock-predicted binding energies of bilirubin and phycocyanobilin with selected protein receptors.

Protein receptor	Bilirubin (kcal/mol)	Phycocyanobilin (kcal/mol)
**EGFR binding site 1 (W2R)**	−9.7	−9.3
**EGFR binding site 2 (SO**_**4**_)	−9.5	−10.2
**EGFR binding site 3 (SO**_**4**_)	−5.5	−8.0
**FYN**	−8.8	−9.5
**HLA-B**	–8.5	–8.8
**LCK**	−9.0	−9.3
**LYN**	−9.2	−10.0
**TP53**	−7.5	−6.6

More negative values indicate stronger predicted binding affinity.

**Fig 1:**
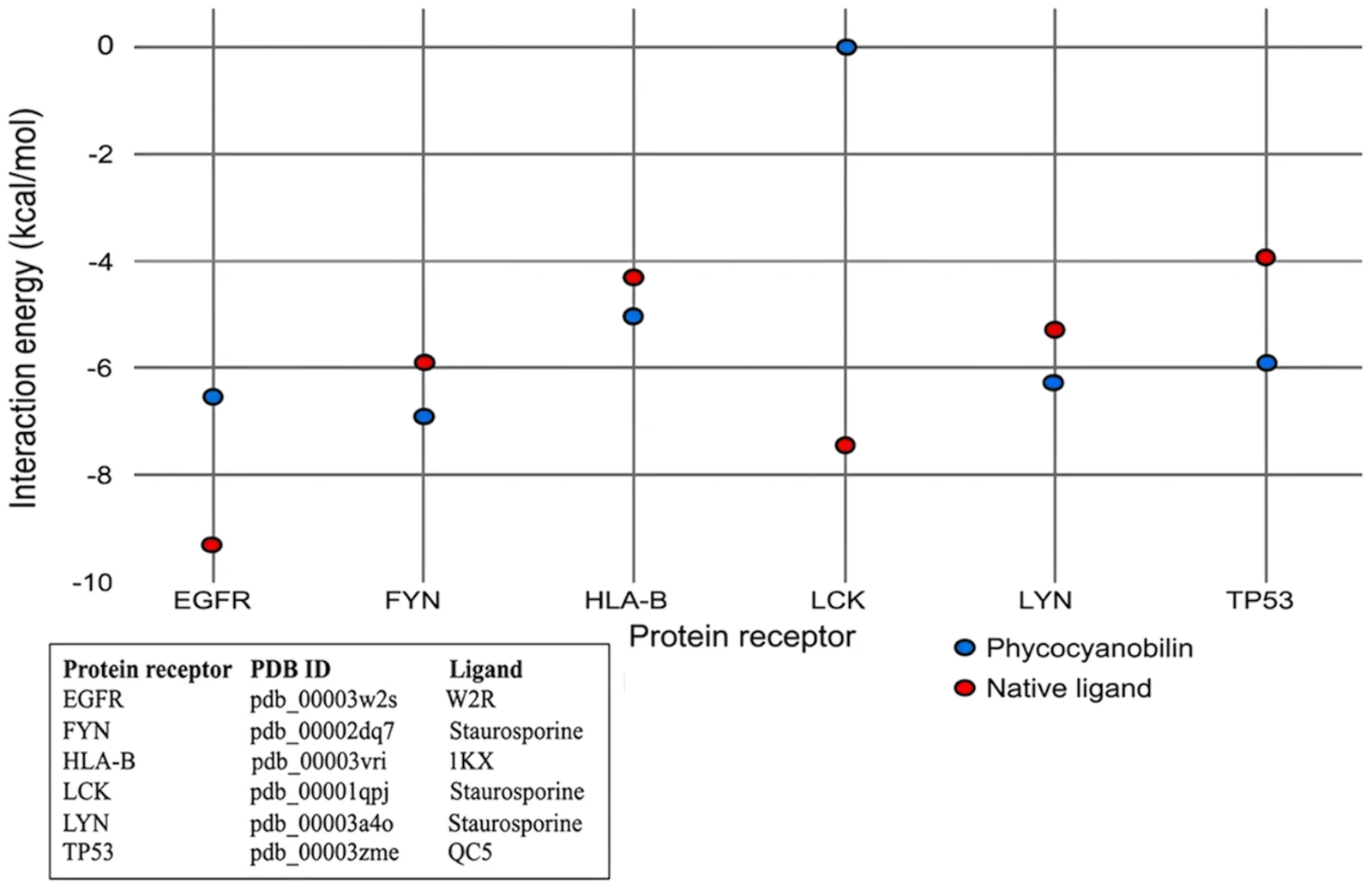
Comparison of docking interaction energies between phycocyanobilin and native ligands across protein receptors. Binding energies (kcal/mol) predicted by AutoDock are shown for phycocyanobilin and the corresponding native ligands obtained from experimentally resolved crystal structures in the Protein Data Bank (PDB). More negative values indicate stronger predicted binding affinity. Phycocyanobilin exhibits more favorable binding energies than the native ligands for FYN, HLA-B, LYN, and TP53.

The docking results of Spirulina compounds with potential targets were compared with those of native ligands from experimentally resolved crystal structures retrieved from the PDB. The results showed that FYN, HLA-B, LYN, and TP53 exhibited more favorable binding energies with phycocyanobilin than with their respective native ligands (Fig 1). To further strengthen the target selection process, we performed KEGG pathway enrichment analysis. Pathways were mapped using the KEGG database, and enrichment was assessed using a hypergeometric test. The selected proteins were significantly enriched in immune-related pathways, including T-cell receptor signaling, NF-kappa B signaling, Fc epsilon RI signaling, and natural killer cell-mediated cytotoxicity (Figure in S3 Fig). These findings further support the biological relevance of the selected targets in immune regulation and SLE pathogenesis.

### Possible targets of phycocyanobilin identified by NetPredictor

Some Spirulina compounds have few or no known targets available for analysis. Therefore, we used NetPredictor, based on network-based inference, to predict potential protein targets for phycocyanobilin. The predicted protein targets of phycocyanobilin, ranked according to their scores, were as follows: PHF1, LYN, WDFY4, TNFSF4, AIF1, TNFSF11, ERCC2, TRIM31, MERTK, and NCR3 (Table 2). Interestingly, LYN kinase was consistently identified as a predicted target, which is in agreement with the molecular docking results (Figure in S4 Fig and Table in S1 Table). The *in silico* prediction analysis further supports the potential of LYN as a key protein target for phycocyanobilin. The identification of LYN as a primary target was consistently supported by both analyses, leading to its selection as the most promising protein receptor for further investigation using MD simulations.

**Table 2:** Protein targets of phycocyanobilin identified using NetPredictor.

Protein	Score	Interaction type
**MAPK14**	0.814752279	Known interaction
**PHF1**	0.000600931	Predicted interaction
**LYN**	0.000579744	Predicted interaction
**WDFY4**	0.000575699	Predicted interaction
**TNFSF4**	0.000568789	Predicted interaction
**AIF1**	0.000550624	Predicted interaction
**TNFSF11**	0.000545884	Predicted interaction
**ERCC2**	0.000543534	Predicted interaction
**TRIM31**	0.000535494	Predicted interaction
**MERTK**	0.000526628	Predicted interaction
**NCR3**	0.000523868	Predicted interaction

Predicted interactions indicate computationally inferred associations, whereas known interactions indicate previously reported or experimentally validated interactions. Scores represent the confidence of predicted interactions and range between 0 and 1, with higher values indicating greater confidence.

### MD simulations of phycocyanobilin and the potential protein target LYN

**Fig 2:**
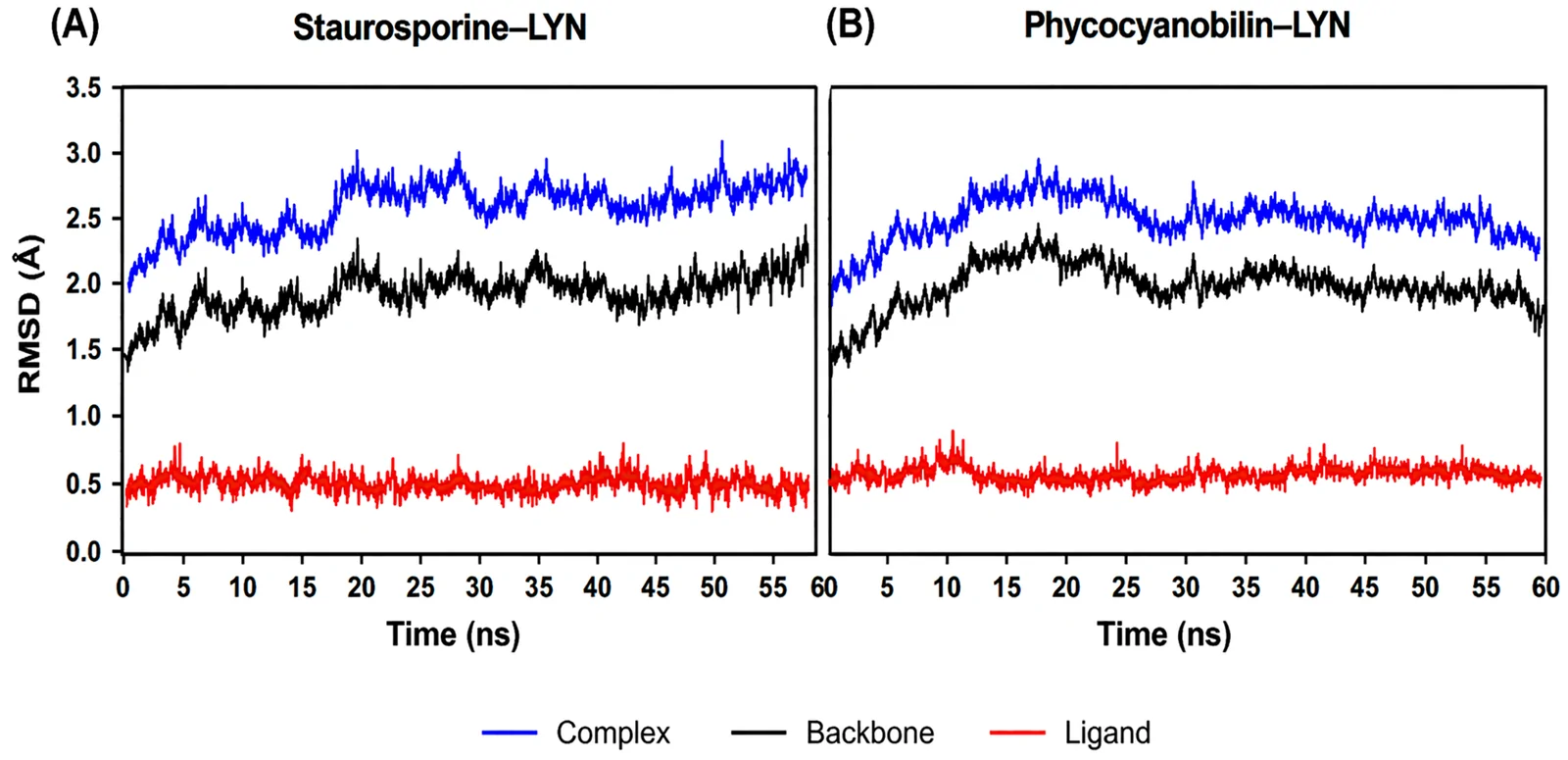
Root-mean-square deviation (RMSD) analysis of LYN-ligand complexes during molecular dynamics simulations. RMSD plots of the protein–ligand complex (blue), protein backbone (black), and ligand (red) are shown for the staurosporine–LYN (A) and phycocyanobilin–LYN (B) systems over a 60 ns molecular dynamics simulation.

Comparative MD simulations were conducted for phycocyanobilin in complex with LYN kinase, alongside the native ligand, staurosporine (PDB ID: 3A4O), a potent inhibitor of protein kinase C. To evaluate the stability of the simulated model, the RMSD of the protein backbone, complex, and ligand was calculated. The results shown in Fig 2 illustrate the RMSD values of the complex (blue), backbone (black), and ligand (red) for the phycocyanobilin–LYN system, in comparison with the staurosporine–LYN complex. The RMSD values of both complexes remained within ~2.5–3 Å, indicating overall structure stability. The RMSD of the phycocyanobilin complex exhibited minor fluctuations during 10–20 ns and reached equilibrium at approximately 27 ns. In addition, the RMSD of the backbone followed a similar fluctuation pattern to that of the complex, suggesting coordinated structural behavior. The RMSD of the phycocyanobilin ligand was relatively more stable than that of staurosporine, and the trajectories of both systems showed comparable trends over the simulation period.

Calculations using the MM/PBSA and MM/GBSA methods yielded consistent estimates of binding free energy. Electrostatic and vdW interactions were the major contributors to the non-covalent binding energy. As shown in Table 3, the non-covalent interaction energy of phycocyanobilin (−98.81 ± 5.84 kcal/mol) was lower than that of the native ligand, staurosporine (−77.33 ± 5.33 kcal/mol), suggesting a trend toward more favorable binding of phycocyanobilin to LYN. The calculated binding free energy (ΔG_bind) of phycocyanobilin (−39.42 ± 8.41 kcal/mol) was likewise more favorable than that of staurosporine (−33.24 ± 5.57 kcal/mol).

**Table 3:** Non-covalent interaction energies of two simulated complexes.

Ligand	Electrostatic energy (kcal/mol)	van der Waals energy (kcal/mol)	Total energy (ΔE_MM, kcal/mol)	Binding free energy (ΔG_bind, kcal/mol)
**Phycocyanobilin**	−25.81 ± 4.98	−73.01 ± 3.34	−98.81 ± 5.84	−39.42 ± 8.41
**Staurosporine**	−25.08 ± 3.38	−52.25 ± 3.48	−77.33 ± 5.33	−33.24 ± 5.57

Interaction energies are presented as mean ± standard deviation and were calculated using the MM/GBSA method over the last 10 ns of a 60 ns molecular dynamics simulation. Total energy (ΔE_MM) represents the sum of electrostatic and van der Waals interaction energies. More negative values indicate stronger non-covalent interactions and binding affinity.

**Fig 3:**
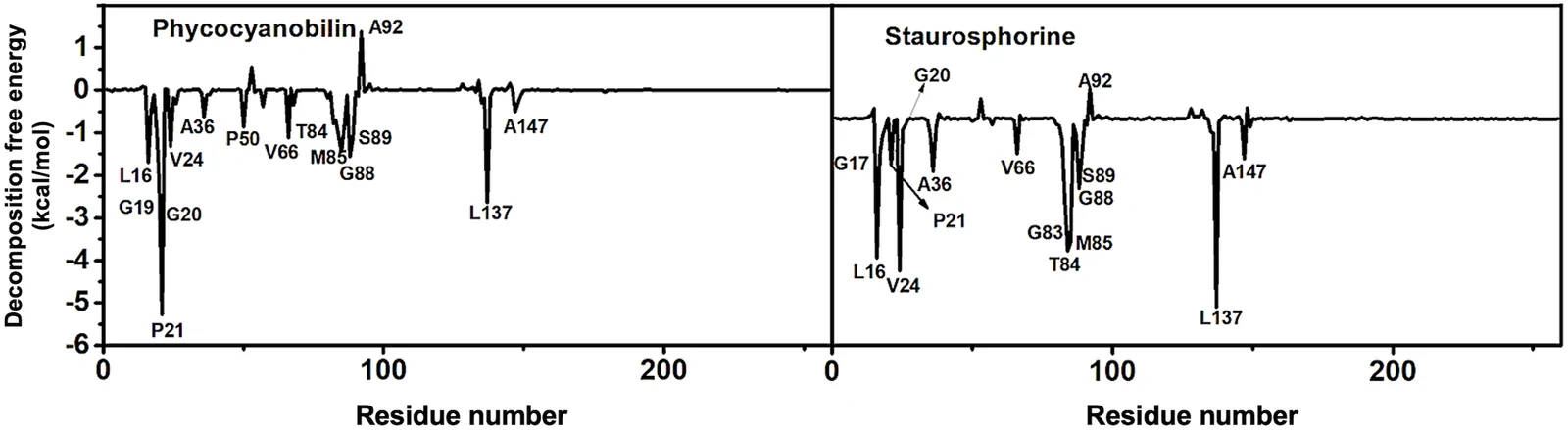
Per-residue decomposition of binding free energy for the phycocyanobilin–LYN and staurosporine–LYN complexes. Negative values indicate stabilizing contributions, whereas positive values indicate destabilizing contributions to ligand binding.

To determine the contribution to the total free energy, per-residue binding free energy decomposition was calculated using the MM/PBSA method. The contribution of each amino acid residue to protein–ligand binding is shown in Fig 3. Phycocyanobilin and staurosporine share several overlapping residues that contribute to binding free energy. More than 10 residues (16–17, 20–21, 24, 36, 66, 84–85, 88–89, 137, 147) were identified as contributing to ligand binding stability in both complexes. Among these, P21, V24, G88, and L137 exhibited the highest contributions to the total free energy. In addition, P21, T84, M85, and L137 were identified as key residues contributing to binding stabilization. Most of the shared residues between phycocyanobilin and staurosporine contributed favorably to ligand binding stability. Notably, P21 showed the strongest stabilizing effect in the phycocyanobilin complex, with a contribution of −5.27 kcal/mol, indicating a significant role in binding affinity.

**Fig 4:**
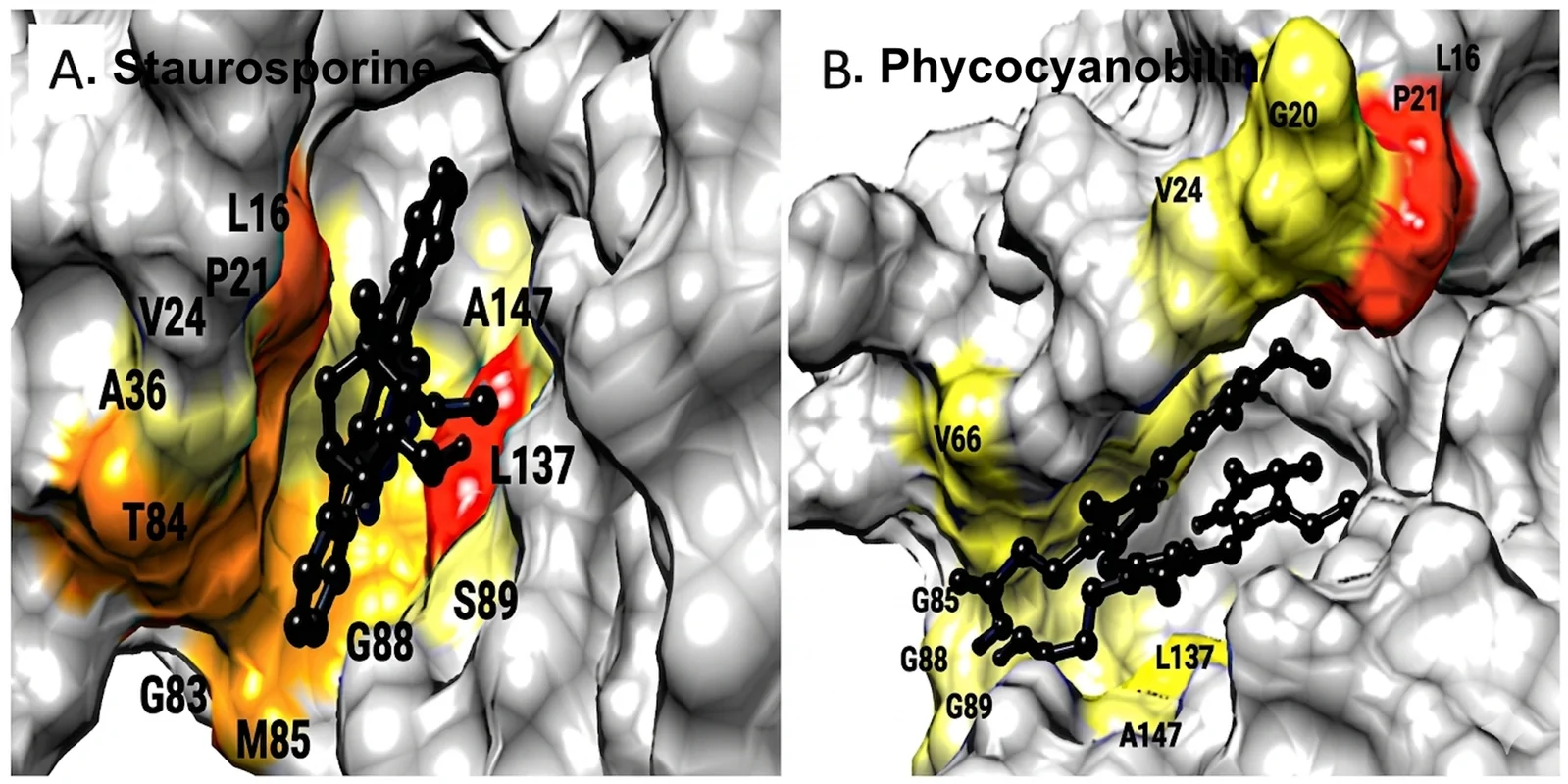
Binding orientation of staurosporine and phycocyanobilin in the LYN kinase active site. (A) Binding orientation of staurosporine and (B) phycocyanobilin from the final snapshot of the molecular dynamics simulation. The protein surface is shown with energy-based coloring (red, lowest energy; yellow, intermediate; gray, highest energy). Key interacting residues are labeled. Molecular graphics and analyses were performed using UCSF Chimera [24], developed by the Resource for Biocomputing, Visualization, and Informatics at the University of California, San Francisco, with support from NIH P41-GM103311.

**Fig 5:**
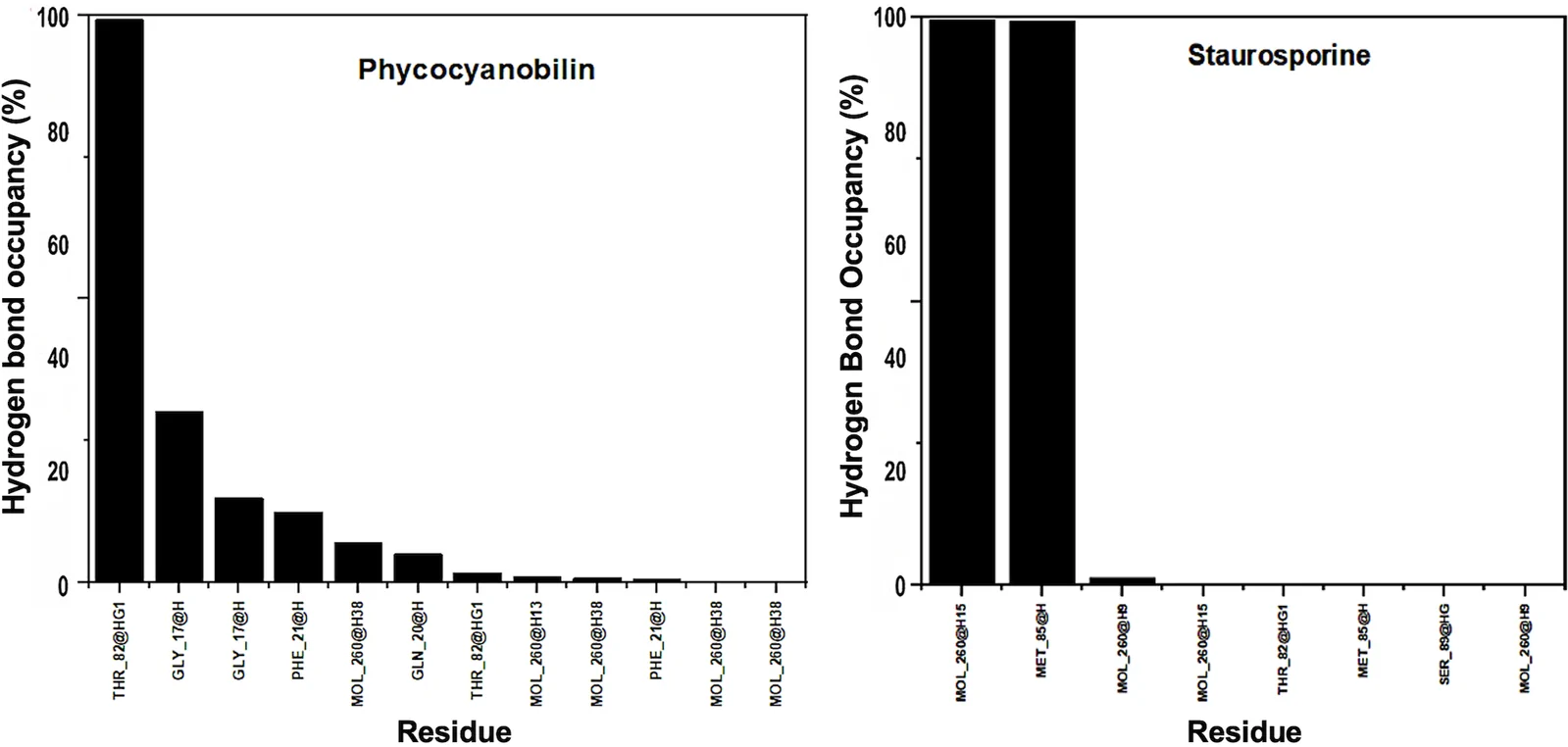
Percentage of hydrogen bond occupancy for amino acid residues contributing to ligand binding in the phycocyanobilin–LYN and staurosporine–LYN complexes. Hydrogen bond occupancy (%) was calculated over the last 10 ns of a 60 ns molecular dynamics simulation.

The binding orientation of phycocyanobilin and staurosporine is displayed in Fig 4. Hydrogen bond formation is a critical factor influencing the binding strength of protein–ligand complexes. The hydrogen bond interactions were evaluated by measuring the distance between hydrogen donor and acceptor atoms. For the phycocyanobilin–LYN complex, one strong hydrogen bond (>90% occupancy) with residue T82 was identified, contributing to binding stabilization. In contrast, the staurosporine–LYN complex exhibited two strong hydrogen bonds (>90% occupancy), involving residues M85 and M260, which also contributed to binding stability (Fig 5). Additional lower-occupancy hydrogen bond interactions were observed in both complexes.

**Fig 6:**
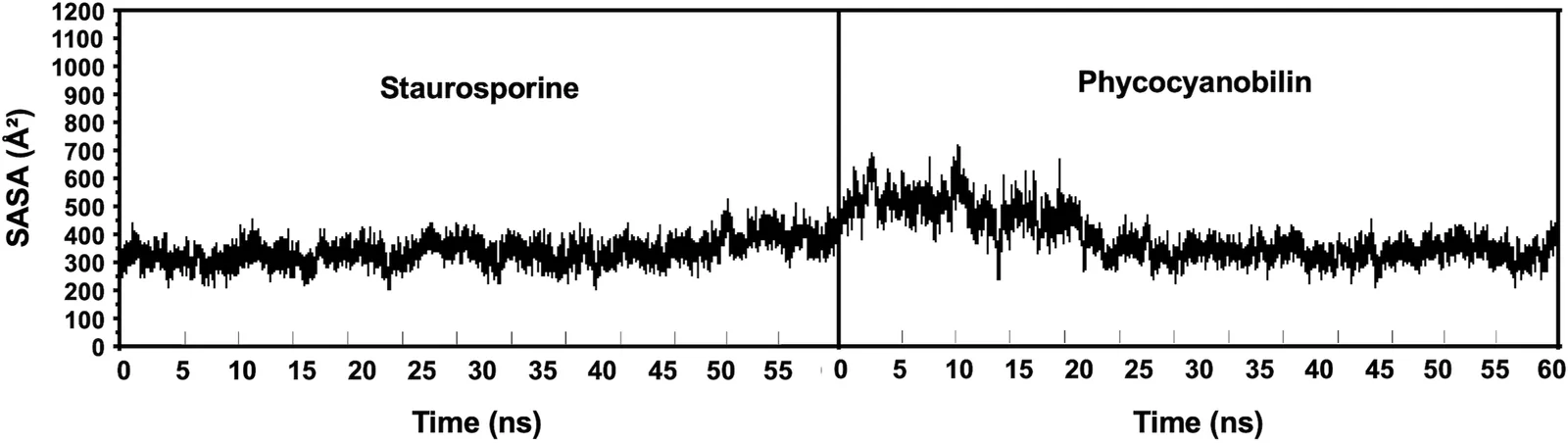
Solvent-accessible surface area (SASA) of the staurosporine–LYN and phycocyanobilin–LYN complexes during molecular dynamics simulations. SASA values (Å²) of surface-exposed residues surrounding the ligands were calculated over a 60 ns molecular dynamics simulation.

To investigate the effect of solvent accessibility on Spirulina compound binding with potential SLE-related targets, the solvent accessible surface area (SASA) was calculated based on surface-exposed amino acid residues around the ligands. As shown in Fig 6, the SASA values of phycocyanobilin and staurosporine exhibited similar trends, ranging within ~250–500 Å^2^. The SASA profiles stabilized and overlapped within ~250–350 Å^2^ after 20 ns and remained stable until the end of the simulation. Therefore, the SASA of phycocyanobilin remained consistently stable at ~300 Å^2^, suggesting stable solvent exposure and ligand binding behavior. Correspondingly, phycocyanobilin exhibited favorable binding affinity toward its potential targets.

**Fig 7:**
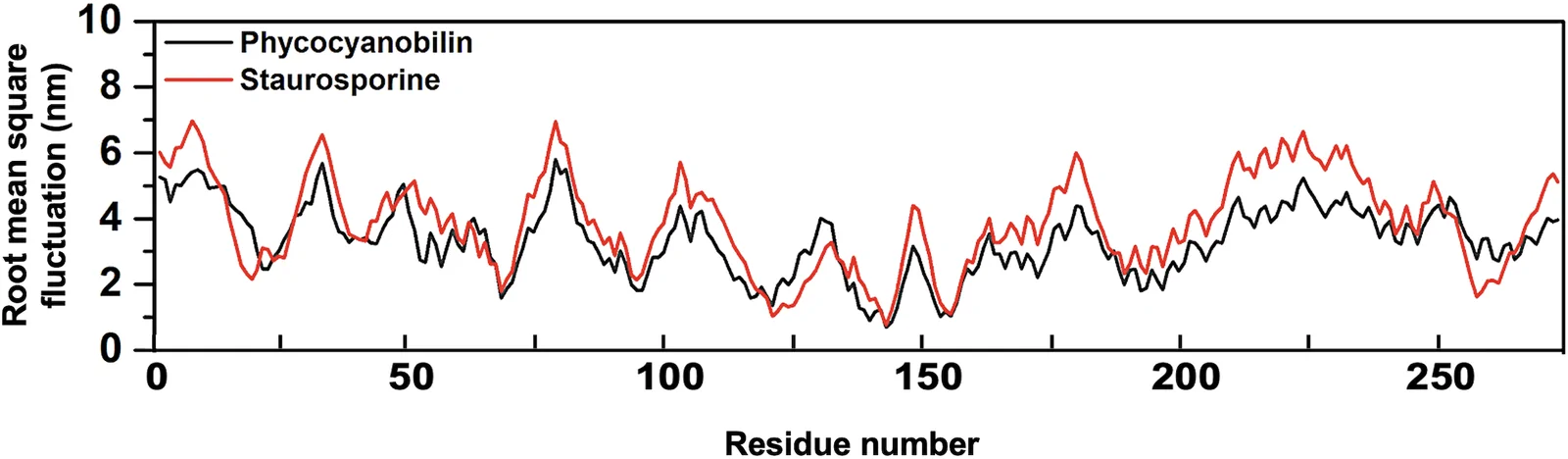
Backbone root-mean-square fluctuation (RMSF) profiles of the phycocyanobilin–LYN and staurosporine–LYN complexes. RMSF values (nm) of protein backbone residues were calculated over a 60 ns molecular dynamics simulation.

Lastly, protein flexibility was calculated using root-mean-square fluctuation (RMSF) analysis of backbone residues. The RMSF profile of the Spirulina compound (phycocyanobilin) compared with the native ligand (staurosporine) is shown in Fig 7. On average, phycocyanobilin exhibited lower fluctuations than staurosporine, indicating that it forms a more stable complex with the LYN protein, likely due to favorable interactions within the active site. However, in the residue regions of ~125–145 and ~250–255, higher fluctuations were observed compared to staurosporine, suggesting that these regions exhibit increased local flexibility compared with the native complex.

## Discussion

In SLE patients, oxidative stress increases with disease activity. Previous studies have shown that serum bilirubin levels are negatively correlated with disease activity. Reduced bilirubin levels in SLE may be associated with inflammation and lupus-related renal involvement [3–5]. Bilirubin likely plays an important role in controlling oxidative stress, and its reduction may result from increased consumption under conditions of severe oxidative stress. Structural similarity analysis revealed a high Tanimoto score of 93% between phycocyanobilin and bilirubin. Both compounds share a high degree of structural similarity as tetrapyrrole chromophores. According to PubChem, a Tanimoto score of ≥90% indicates a high level of confidence, suggesting that their core structures and key functional groups are sufficiently similar and occupy closely related chemical space. However, structural similarity alone does not predict equivalent biological activity, which requires experimental validation.

In this study, structure–activity analysis demonstrated that phycocyanobilin mimics the binding orientation of bilirubin within the LYN kinase ATP-binding pocket, forming stabilizing hydrogen bonds (i.e., T82). These findings support its role as a functional analog of bilirubin, which is known to protect against SLE by modulating oxidative stress and immune signaling. Consequently, the favorable binding affinity of phycocyanobilin toward LYN suggests that it may interact with LYN and potentially modulate LYN-mediated signaling pathways. Phycocyanobilin is a bioactive compound derived from the metabolic network of *A. platensis* C1. Reported biological activities of phycocyanobilin include inhibition of vascular endothelial growth factor receptor 2, expansion of regulatory T cells, and activation of the aryl hydrocarbon receptor, all of which are relevant to SLE pathogenesis. Additionally, phycocyanobilin exhibits antioxidation, anti-inflammation, anti-atherosclerotic, and immunomodulatory effects. It enhances host defense against infectious diseases by supporting mucosal immune function and reduces allergic inflammation through suppression of antigen-specific IgE antibodies [25]. From the PubChem database, phycocyanobilin is associated with eight bioactivity-related compounds, including bilirubin (CID 5280352), biliverdine (CID 5280353), NSC635691 (CID 11606751), biliverdine (CID 6183357), pheophytin (CID 21252250), CHEMBL436528 (CID 44420557), CHEMBL374422 (CID 44420558), and an algal-derived compound (CID 54611329). Based on molecular docking results, LYN, LCK, FYN, and EGFR exhibited favorable binding energies with phycocyanobilin. In addition, NetPredictor identified LYN among the top predicted protein targets of phycocyanobilin, along with PHF1, WDFY4, TNFSF4, AIF1, TNFSF11, ERCC2, TRIM31, MERTK, and NCR3. The consistent identification of LYN across both approaches highlights its potential as a key protein target for phycocyanobilin. To validate the docking protocol, the native ligand (staurosporine) was re-docked into the LYN kinase active site (PDB ID: 3A4O). The resulting RMSD value, maintained within ~2.5–3 Å, indicates that the docking procedure accurately reproduces the experimental binding mode, thereby supporting the reliability of the docking results. This structural consistency was further supported by MD simulations, in which the complex achieved equilibrium at 27 ns while maintaining a stable binding orientation. Additionally, phycocyanobilin demonstrated a more favorable binding free energy (ΔG_bind) of –39.42 ± 8.41 kcal/mol compared to staurosporine’s –33.24 ± 5.57 kcal/mol, driven by significantly stronger non-covalent interaction energies (–98.81 vs. –77.33 kcal/mol). This difference may be biologically significant, as LYN kinase functions as a key negative regulator of B-cell signaling. The enhanced affinity is attributed to stable hydrogen bonding and hydrophobic interactions with key residues, including P21, T84, and M85, within the ATP-binding pocket. These findings suggest that phycocyanobilin occupies the LYN active site with a stability comparable to or greater than that of known high-affinity inhibitors, supporting its potential as a bioactive modulator of LYN kinase signaling in SLE. LYN, a member of the Src family of tyrosine kinases, plays a crucial role in regulating immune responses, B-cell differentiation, apoptosis, and cytokine signaling pathways. Depending on the context, LYN can function as either an inhibitor or an activator [26]. Loss of LYN function results in a lupus-like autoimmune phenotype characterized by hyperactive B cells and myeloproliferation. This is supported by LYN-/- mouse models, which develop SLE-like disease with plasma cell hyperplasia [27]. In humans, LYN expression is inversely associated with disease activity, albuminuria levels, and renal pathology in SLE [28].

Previous studies have explored bioactive compounds from Spirulina with immunomodulatory effects; however, they did not specify individual compounds, instead examining Spirulina as whole extracts. While earlier work, including our own, employed network-based approaches to identify potential bioactive compounds from Spirulina, the present study provides novel mechanistic insight by specifically investigating the interaction between phycocyanobilin and LYN kinase. Unlike prior studies, we incorporated molecular docking, MD simulations, and binding free energy analyses, revealing the stability, binding mode, and key residue-level interactions of the phycocyanobilin–LYN complex. Furthermore, comparison with the native ligand strengthens the functional relevance of our findings. Collectively, these approaches provide detailed structural and mechanistic insight beyond association-based predictions. Therefore, bioactive compounds that modulate LYN activity may hold significant potential for targeting SLE-associated immune dysregulation.

To evaluate the suitability of phycocyanobilin as a therapeutic candidate for SLE, an *in silico* assessment was conducted using Lipinski’s Rule of Five (RO5) [29], ADMET profiling, and related drug-likeness parameters. Phycocyanobilin has a molecular weight (MW) of 586.68 Da, a lipophilicity (LogP) of 2.49, a topological polar surface area (TPSA) of 164.71 Å², ten rotatable bonds, five hydrogen bond donors, and seven hydrogen bond acceptors. Its relatively high MW and TPSA suggest that passive membrane permeability and oral absorption may be lower than those of compounds that fully satisfy Lipinski's RO5. Nevertheless, these properties do not preclude its potential as a lead compound, as many natural products possess physicochemical characteristics outside conventional drug-likeness criteria while retaining biological activity. Further lead optimization or formulation strategies may improve its pharmacokinetic properties. Importantly, its lipophilicity remains within the acceptable range (LogP < 5), supporting further development. ADMET profiling using ADMETlab 3.0 [30] predicts that, despite low membrane permeability, phycocyanobilin exhibits adequate human intestinal absorption and favorable oral bioavailability (F20%, F30%, and F50%). It also demonstrates good natural product–likeness (NP score = 0.714), supporting its potential as a bioactive lead. Regarding safety, phycocyanobilin is derived from *A. platensis* (Spirulina), which has a well-established history of safe human consumption. Toxicity prediction using ProTox-3.0 [31] classifies phycocyanobilin as toxicity class 5, with a predicted LD₅₀ of 3,066 mg/kg, indicating relatively low acute toxicity. Although its physicochemical properties suggest room for optimization, the favorable predicted safety profile, strong binding affinity toward LYN kinase, and structural similarity to endogenous bilirubin support its promise as a lead compound for SLE drug development. Nevertheless, these findings are based solely on computational predictions and require experimental validation. The function of LYN in immune regulation is context-dependent. Although phycocyanobilin exhibited stable binding to the LYN kinase domain in the present study, future studies should include kinase selectivity profiling, *in vitro* kinase assays, and cell-based experiments (e.g., immune signaling and B-cell activation assays) to confirm its biological relevance and target specificity of phycocyanobilin in SLE.

## Conclusions

Serum bilirubin levels in patients with SLE are negatively correlated with disease activity. Phycocyanobilin, a bioactive compound derived from *Arthrospira platensis* C1, showed high structural similarity to bilirubin (93%). Multiple complementary computational approaches, including molecular docking, target prediction, pathway enrichment, molecular dynamics simulations, and binding free energy analyses, consistently identified LYN as a biologically relevant target and demonstrated stable binding of phycocyanobilin to LYN. Collectively, these findings suggest that phycocyanobilin warrants further investigation as a potential modulator of LYN-associated signaling pathways in SLE.

## Supporting information

S1 Fig Structural similarity between phycocyanobilin and bilirubin. Two-dimensional chemical structures of phycocyanobilin (CID: 137699530) and bilirubin (CID: 5280352) were obtained from the PubChem database (https://pubchem.ncbi.nlm.nih.gov). The high structural similarity between the two compounds is supported by a Tanimoto score of 93%.
(TIF)

S2 Fig Comparison of molecular docking results of phycocyanobilin with selected protein receptors using AutoDock, FlexX, and iGEMDOCK. Docking scores were generated using the native scoring function implemented in each docking program and therefore should not be directly compared numerically across different software platforms.
(TIFF)

S3 Fig KEGG pathway enrichment analysis of the selected protein targets (EGFR, FYN, HLA-B, LCK, LYN, and TP53). Bubble plot showing significantly enriched KEGG pathways for the selected protein targets. Bubble size represents the number of associated genes, and color indicates the enrichment *Q* value.
(TIFF)

S4 Fig Visualization of the predominant binding mode of phycocyanobilin in the LYN kinase active site. The protein is shown as a surface representation, with phycocyanobilin displayed in stick form within the binding pocket. Molecular graphics and analyses were performed using UCSF Chimera [24], developed by the Resource for Biocomputing, Visualization, and Informatics at the University of California, San Francisco, with support from NIH P41-GM103311.
(TIF)

S1 Table AutoDock-predicted binding modes of phycocyanobilin (ligand) with LYN (PDB ID: 3A4O). (PDF)

## Abbreviations

SLE: systemic lupus erythematosus
CID: compound identifier
