# Supplementary material for: Phycocyanobilin: A potential bioactive compound from Arthrospira platensis targeting LYN protein associated with systemic lupus erythematosus
Source: PLoS One. 2026 Aug 28;21(8):e0357093. doi: 10.1371/journal.pone.0357093 (PMC13524268; doi:10.1371/journal.pone.0357093)
Supplement: Supplementary file 1 [file pone.0357093.s005.pdf]

S1 Table. AutoDock-predicted binding modes of phycocyanobilin with LYN (PDB ID: 3A4O).

**Mode**	**Affinity (kcal/mol)**	**RMSD (lower bound, Å)**	**RMSD (upper bound, Å)**
**1**	−10.0	0.000	0.000
**2**	−10.0	1.846	2.591
**3**	−9.8	1.456	2.410
**4**	−9.8	3.221	9.998
**5**	−9.8	3.503	6.501
**6**	−9.7	2.494	9.708
**7**	−9.7	1.523	10.312
**8**	−9.4	1.394	1.812
**9**	−9.3	3.144	6.435

Values represent the top-ranked docking poses generated by AutoDock using MGLTools.

Lower (more negative) binding energy indicates stronger predicted binding affinity.

RMSD, root mean square deviation.
