# Supplementary material for: Phycocyanobilin: A potential bioactive compound from Arthrospira platensis targeting LYN protein associated with systemic lupus erythematosus
Source: PLoS One. 2026 Aug 28;21(8):e0357093. doi: 10.1371/journal.pone.0357093 (PMC13524268; doi:10.1371/journal.pone.0357093)
Supplement: Supplementary file 2 [file pone.0357093.s006.pdf]

Dr. Prof. Editor of Journal of *PLOS ONE*

Dear Editor,

Thank you for the review invitation.

Concerning the manuscript;

Ref.; PONE-D-25-50743

I have reviewed a research article entitled "**Phycocyanobilin: A potential bioactive compound from *Arthrospira platensis* targeting LYN protein associated with systemic lupus erythematosus,**" submitted to PLOS ONE by Chaiprasert et al. In general, this manuscript investigates the potential therapeutic role of phycocyanobilin derived from Arthrospira platensis in targeting the LYN protein associated with systemic lupus erythematosus (SLE). The study combines structural similarity analysis, molecular docking, target prediction, and molecular dynamics simulations to identify phycocyanobilin as a promising ligand for LYN kinase. The topic is relevant and timely because SLE remains a complex autoimmune disease with limited targeted therapies. The computational workflow used in this study is appropriate, and the manuscript presents interesting findings suggesting that phycocyanobilin may interact strongly with LYN protein, potentially contributing to immune regulation in SLE. However, several methodological clarifications, structural improvements, and additional discussion are needed before the manuscript can be considered for publication. So, I recommend the manuscript for **major revision**.

Regards,

Comments to the Author

Major Comments

- While the manuscript highlights the potential interaction between phycocyanobilin and LYN kinase, the novelty should be clarified more explicitly. Previous studies have already explored bioactive compounds from Spirulina with immunomodulatory effects. The authors should clearly state: What new mechanistic insight does this study provide? How do their findings differ from the previously published network analysis study referenced in the manuscript?

- The study focuses primarily on LYN after docking against several receptors (EGFR, FYN, HLA-B, LCK, LYN, and TP53). However, the criteria for selecting LYN as the primary target require further explanation. The authors should clarify why LYN was prioritized over FYN or LCK, which also showed favorable docking scores. Were statistical comparisons between docking scores performed?
- The docking procedure requires additional methodological details to ensure reproducibility. The authors should specify the Grid box dimensions and coordinates. Number of docking runs. Exhaustiveness or search parameters used in AutoDock. Validation of the docking protocol (e.g., re-docking of native ligand). Without these details, it is difficult to assess the reliability of the docking results.
- Although MD simulations were conducted for 60 ns, several parameters are missing. The authors should report the temperature and pressure conditions used in simulations. Solvent model applied. Equilibration protocol. Time step used during simulations. Furthermore, a 60 ns simulation may be relatively short for stable protein–ligand interaction analysis. The authors should justify this duration.
- The manuscript reports binding free energies using MM/PBSA and MM/GBSA methods. However, the difference between phycocyanobilin and the native ligand (staurosporine) should be statistically evaluated. Error bars or confidence intervals should be discussed more clearly. The biological relevance of these energy differences should be explained.
- The conclusions suggest that phycocyanobilin could serve as a therapeutic compound for SLE. However, the study relies entirely on in silico approaches. The authors should clearly acknowledge this limitation. Avoid making strong therapeutic claims. Suggest future in vitro or in vivo validation experiments.
- The discussion should better connect computational findings with biological mechanisms of SLE. Explain how LYN modulation affects immune signaling pathways. Discuss how phycocyanobilin may influence B-cell regulation and autoimmune responses. Include more recent literature on LYN signaling in SLE.
- Several grammatical and stylistic issues should be corrected. Examples include:

1. "MD simulations result demonstrates" → should be "MD simulation results demonstrate."
2. "better docking interaction energy" → should be "more favorable docking interaction energy."

3. Professional language editing is recommended.

- The abstract contains excessive numerical details. It would benefit from shortening technical descriptions. Highlighting the main biological implication.
- Figures need improvement: Axis labels should be clearer in RMSD, RMSF, and SASA plots. Figure captions should describe the results more completely.
- Several references contain formatting inconsistencies and duplicated entries (e.g., bilirubin signaling references). The reference list should be carefully checked in accordance with the journal's guidelines.
