# Supplementary material for: Phycocyanobilin: A potential bioactive compound from Arthrospira platensis targeting LYN protein associated with systemic lupus erythematosus
Source: PLoS One. 2026 Aug 28;21(8):e0357093. doi: 10.1371/journal.pone.0357093 (PMC13524268; doi:10.1371/journal.pone.0357093)
Supplement: Supplementary file 3 [file pone.0357093.s007.pdf]

Response to Editor's and Reviewers' comments

PONE-D-25-50743
"Phycocyanobilin: A potential bioactive compound from *Arthrospira platensis* targeting LYN protein associated with systemic lupus erythematosus"

Editor

----

1. Figure 4, S2, S3, in the submission contain copyrighted images.

Response:

- S2 Fig has been removed as it contained copyrighted material obtained from the PDB.
    - S2 Fig. Structure of the receptor protein, EGFR kinase, domain with three binding sites (W2R and two SO4 binding sites) in PDB format retrieved from RCSB PDB (https://www.rcsb.org).
- Fig 4 and S3 Fig are retained as they were generated in this study using UCSF Chimera. To ensure compliance with Chimera usage guidelines, the following revisions have been made: (i) Appropriate acknowledgment and citation of UCSF Chimera have been added to the Fig 4 caption and the S2 Fig caption (renumbered), and (ii) the figure numbering has been updated accordingly (the former S3 → updated S2, Line 172).
    - Line 247-254: "**Fig 4. Binding orientation of staurosporine and phycocyanobilin in the LYN kinase active site.** (A) Binding orientation of staurosporine and (B) phycocyanobilin from the final snapshot of the molecular dynamics simulation. The protein surface is shown with energy-based coloring (red, lowest energy; yellow, intermediate; gray, highest energy). Key interacting residues are labeled. Molecular graphics and analyses were performed using UCSF Chimera [24], developed by the Resource for Biocomputing, Visualization, and Informatics at the University of California, San Francisco, with support from NIH P41-GM103311."
    - Line 508-513: "**S2 Fig. Visualization of the predominant binding mode of phycocyanobilin in the LYN kinase active site.** The protein is shown as a surface representation, with phycocyanobilin displayed in stick form within the binding pocket. Molecular graphics and analyses were performed using UCSF Chimera [24], developed by the Resource for Biocomputing, Visualization, and Informatics at the University of California, San Francisco, with support from NIH P41-GM103311."

----

2. Is the manuscript presented in an intelligible fashion and written in standard English?
Reviewer #1: Yes
Reviewer #2: No

Response:

- The manuscript has been revised for clarity and adherence to standard academic English. Grammar, sentence structure, and terminology have been carefully edited throughout the text. The manuscript has also been improved for readability, consistency, and overall presentation.

Reviewer #1

---

1. The study relies solely on *in silico* predictions. Authors should clearly acknowledge this limitation and suggest experimental validation (*in vitro* kinase assay or cell-based validation).

Response:

- Our study is based on *in silico* approaches, which inherently have limitations. The manuscript has been revised to explicitly acknowledge this in the Discussion section.
- Line 357-359: "However, this study is based entirely on *in silico* approaches, including molecular docking, target prediction, and MD simulations, which have inherent limitations in predicting biological activity in complex physiological systems."
- Line 378-381: "Nevertheless, experimental validation is required. Future studies should include *in vitro* kinase assays and cell-based experiments (e.g., immune signaling or B-cell activation assays) to confirm its biological relevance in the context of SLE."

---

2. Docking protocol validation is not clearly described. Provide re-docking of the native ligand and report RMSD values to validate the docking protocol.

Response:

- As suggested by the reviewer, the docking protocol validation has been clarified, and the following text has been added or revised:
- Line 102-107: "The reliability of the docking protocol was validated by comparing the predicted binding energies of the studied compounds with those of native ligands from experimentally resolved crystal structures available in the PDB, using the same grid parameters and search settings. The accuracy of the protocol was confirmed by calculating the root-mean-square deviation (RMSD) between the predicted docking poses and the original crystal structures."
- Line 323-329: "To validate the docking protocol, the native ligand (staurosporine) was re-docked into the LYN kinase active site (PDB ID: 3A4O). The resulting RMSD value, maintained within ~2.5–3 Å, indicates that the docking procedure accurately reproduces the experimental binding mode, thereby supporting the reliability of the docking results. This structural consistency was further supported by MD simulations, in which the complex achieved equilibrium after 27 ns while maintaining a stable binding orientation."

---

3. Lack of detailed docking parameters. Include grid box dimensions, scoring function, exhaustiveness, and docking software settings.

Response:

- Detailed docking parameters have been revised and added to the Materials and Methods and Results sections.
- Line 97-107: "To ensure comprehensive coverage of potential binding sites, the grid box was configured to encompass the relevant binding domains of each receptor, enabling thorough

sampling of potential binding conformations. For the EGFR kinase domain, the grid box specifically included the W2R binding site and two SO4 sites. Detailed grid coordinates and exhaustiveness parameters were defined for each AutoDock run. The reliability of the docking protocol was validated by comparing the predicted binding energies of the studied compounds with those of native ligands from experimentally resolved crystal structures available in the PDB, using the same grid parameters and search settings. The accuracy of the protocol was confirmed by calculating the root-mean-square deviation (RMSD) between the predicted docking poses and the original crystal structures."

- Line 144-147: "The grid box was configured to encompass these binding sites, after which bilirubin and phycocyanobilin were docked to each receptor. The AutoDock results for both ligands, bilirubin and phycocyanobilin, against the selected receptors, as obtained using MGLTools, are summarized in Table 1."

----

4. Limited comparison with known inhibitors of LYN. Compare phycocyanobilin with established LYN kinase inhibitors to contextualize binding affinity.

Response:

- Our results (Fig 3-7, Table 3) have shown the binding behavior of **phycocyanobilin** to LYN, compared with **staurosporine**, a well-established kinase inhibitor used as the native ligand for LYN (PDB ID: 3A4O). Phycocyanobilin exhibited a more favorable binding free energy (ΔG_bind) than staurosporine and showed comparable interaction patterns within the ATP-binding pocket, including key residues such as P21, T84, and M85. These results provide a relevant reference point for interpreting binding affinity and suggest that phycocyanobilin may achieve a binding stability comparable to that of a known inhibitor. [Line 221-231; 237-245; 261-269; 276-283]

----

5. Binding site analysis is insufficient. Provide detailed analysis of key amino acid residues involved in binding, hydrogen bonds, hydrophobic interactions, and π-interactions.

Response:

- The binding site analysis has been revised to better describe the residue-level interactions involved in ligand binding. The revised manuscript includes per-residue binding free energy decomposition, identification of key residues contributing to complex stability, and hydrogen bond occupancy analysis during the MD simulation. Residues such as P21, T84, M85, and L137 were identified as major contributors to the stability of the phycocyanobilin–LYN complex, and a strong hydrogen bond with T82 was also observed. Hydrophobic interactions within the ATP-binding pocket were also described. π-interactions were not specifically quantified in the current analysis and therefore were not overinterpreted; however, the revised text provides a clearer residue-level description of the main stabilizing interactions observed in the complex. [Line 221-231; 237-245]

----

6. Protein structure preparation not fully described. Clarify how the LYN protein structure was prepared (removal of water molecules, protonation states, energy minimization).

Response:

- The preparation of the protein structure has been clarified, and the following text has been added or revised:
- Line 112-118: "The LYN kinase structure (PDB ID: 3A4O) was prepared by removing all water molecules and heteroatoms to obtain the apo-protein. Protonation states of all ionizable amino acid residues were assigned at pH 7.0 using PROPKA 3.0 to simulate physiological conditions. The structure was then subjected to energy minimization using the sander module in AMBER 16 to resolve steric clashes and optimize geometry. MD simulations were performed for 60 ns using AMBER 16, with the ff14SB force field applied to both the protein and ligand."

----

7. MD simulation duration may be insufficient. Extend MD simulations or justify simulation length to ensure system stability and reliability.

Response:

- The simulation length has been clarified and justified in the revised manuscript. A 60 ns molecular dynamics simulation was performed for the phycocyanobilin–LYN complex and the native ligand complex, and system stability was assessed using multiple parameters, including RMSD, SASA, RMSF, hydrogen bond occupancy, and binding free energy analysis. The RMSD profile showed that the phycocyanobilin–LYN complex reached equilibrium after approximately 27 ns and remained stable for the rest of the simulation, while the final 10 ns of the trajectory were used for binding free energy and interaction analyses. These results support that the selected simulation length was sufficient to evaluate complex stability and binding behavior in this study.

----

8. Trajectory analysis is limited. Include additional MD parameters such as RMSF, radius of gyration (Rg), SASA, and hydrogen bond analysis.

Response:

- The 60 ns MD trajectory was thoroughly analyzed using several key parameters to ensure the structural integrity and stability of the phycocyanobilin–LYN complex. RMSF analysis revealed that phycocyanobilin exhibited lower average atomic fluctuations compared to the native inhibitor staurosporine, particularly within the binding pocket residues. The Radius of Gyration (Rg) remained consistently stable throughout the production run, indicating that the protein maintained a compact and well-folded conformation upon ligand binding. SASA values stabilized at approximately 300 $Å^2$ after the initial 20 ns, confirming the steady burial of the ligand within the hydrophobic cavity. Finally, hydrogen bond analysis identified persistent interactions with residues P21, G24, T84, and M85, which provided the primary stabilizing forces for the complex. These combined metrics demonstrate a highly stable and convergent binding profile throughout the simulation.

----

9. Energy decomposition analysis is missing. Perform per-residue energy decomposition to identify residues contributing most to ligand binding.

Response:

- Per-residue energy decomposition analysis has been added to identify the amino acid residues contributing most to ligand binding. In the revised manuscript, MM/PBSA-based per-residue decomposition was used to evaluate residue-level contributions to the total binding free energy. The analysis showed that phycocyanobilin and staurosporine share several residues that contribute to binding stability, with P21, V24, G88, and L137 showing the strongest contributions. In addition, P21, T84, M85, and L137 were identified as key residues involved in stabilizing the phycocyanobilin–LYN complex.

----

10. No ADMET or drug-likeness evaluation. Evaluate pharmacokinetic properties such as Lipinski rules, ADMET, toxicity prediction, and bioavailability.

Response:

- We agree that ADMET and drug-likeness evaluation are important; the following analyses have been added:
- Line 362-378: "To evaluate the suitability of phycocyanobilin as a therapeutic candidate for SLE, an *in silico* assessment was conducted using Lipinski's Rule of Five (RO5) [29], ADMET profiling, and related parameters. Phycocyanobilin has a molecular weight (MW) of 586.68 Da, lipophilicity (LogP) of 2.49, a topological polar surface area (TPSA) of 164.71 Å^2, ten rotatable bonds, five hydrogen bond donors, and seven hydrogen bond acceptors. It exceeds the RO5 threshold for molecular weight (>500 Da), representing a minor violation**;** however, its lipophilicity remains within the acceptable range (LogP < 5), supporting its potential for further optimization. ADMET profiling using ADMETlab 3.0 [30] indicates that, despite low predicted membrane permeability, phycocyanobilin demonstrates adequate human intestinal absorption and favorable oral bioavailability (F20%, F30%, and F50**%).** It also exhibits good natural product–likeness (NP score = 0.714), supporting its potential as a bioactive lead. Regarding safety, phycocyanobilin is derived from *A. platensis* (Spirulina), which has a well-established history of safe human consumption. Toxicity prediction using ProTox-3.0 [31] classifies phycocyanobilin as toxicity class 5, with a predicted LD_{50} of 3,066 mg/kg, indicating relatively low acute toxicity. Despite these limitations, its favorable safety profile, strong binding affinity to LYN kinase, and structural similarity to endogenous bilirubin support its potential as a lead compound for SLE drug development."

----

11. Lack of pathway or biological context discussion Discuss how inhibition of LYN may mechanistically influence SLE-related signaling pathways.

Response:

- As suggested by the reviewer, we have explained the role of LYN as a key regulator of immune responses, B-cell differentiation, apoptosis, and cytokine signaling, and to describe how loss of LYN function is associated with lupus-like autoimmunity, hyperactive B cells, and myeloproliferation. We also clarified that the favorable binding of phycocyanobilin to the LYN ATP-binding pocket, along with its stable interactions with key residues, supports its potential to modulate LYN-associated signaling pathways relevant to B-cell regulation and autoimmune

responses in SLE. In addition, the Discussion has been updated to include more recent literature on LYN signaling in SLE, including evidence linking LYN expression with disease activity, albuminuria, and renal pathology (Reference 28). [Line 300-307; 308-314; 335-344]

----

12. Network pharmacology analysis could strengthen the study. Include protein–protein interaction or pathway enrichment analysis to support biological relevance.

Response:

- We agree that network pharmacology analysis could further strengthen the biological interpretation of this study. In the current work, a network-based target prediction approach (NetPredictor) was already employed to identify potential protein targets of phycocyanobilin, which consistently highlighted LYN kinase as a key candidate. However, comprehensive protein–protein interaction or pathway enrichment analyses were beyond the scope of this study. This limitation has been acknowledged in the Discussion section, and we suggest that future studies incorporate these approaches to better elucidate the broader biological pathways and molecular mechanisms underlying the effects of phycocyanobilin in SLE. [Line 378-381]

----

13. Structural similarity to bilirubin is mentioned but not deeply analyzed. Provide a structure-activity comparison and potential functional implications of this similarity.

Response:

- Phycocyanobilin and bilirubin share a high degree of structural homology as tetrapyrrole chromophores. Bilirubin shows a high Tanimoto score (93%) with phycocyanobilin. In PubChem, a Tanimoto score ≥90% between chemically similar compounds indicates a very high level of confidence, suggesting that their core structures and key functional groups are sufficiently similar to produce the same direction of biological activity. In this study, structure–activity analysis shows that phycocyanobilin mimics bilirubin's binding orientation within the LYN kinase ATP-binding pocket, forming stabilizing hydrogen bonds (i.e. T82). This supports its role as a functional analog of bilirubin, which is known to protect against SLE by modulating oxidative stress and immune signaling. Consequently, the high binding affinity of phycocyanobilin toward LYN suggests it may replicate or enhance these therapeutic effects, offering an algal-derived alternative for targeting the LYN-mediated pathways that are dysregulated in SLE patients with low serum bilirubin levels. [Line 133-142; Table 1]

----

14. Figures may lack clarity in interaction visualization. Improve structural visualization showing 2D and 3D interaction maps of ligand–protein complexes.

Response:

- The figures have been revised to enhance interaction visualization. Updated 3D binding views and 2D interaction maps clearly highlight key residues and interactions, improving interpretation of the ligand–protein complexes.

---

15. Statistical robustness of simulation results is unclear. Include replicate simulations or statistical confidence intervals for binding energy calculations.

Response:

- We acknowledge the importance of statistical robustness in simulation studies. In this work, binding free energies were calculated from the MD trajectories using MM/GBSA over the final 10 ns, and results are reported as mean ± standard deviation to reflect variability. While replicate simulations were not performed in the current study, this limitation has been acknowledged in the Discussion section.
- Line 359-364: While replicate MD simulations were not included, the current simulation demonstrated stable and consistent protein–ligand interactions; additional independent runs would further support the robustness of the findings. To evaluate the suitability of phycocyanobilin as a therapeutic candidate for SLE, an *in silico* assessment was conducted using Lipinski's Rule of Five (RO5) [29], ADMET profiling, and related parameters.

Reviewer #2

---

1. While the manuscript highlights the potential interaction between phycocyanobilin and LYN kinase, the novelty should be clarified more explicitly. Previous studies have already explored bioactive compounds from Spirulina with immunomodulatory effects. The authors should clearly state: What new mechanistic insight does this study provide? How do their findings differ from the previously published network analysis study referenced in the manuscript?

Response:

- The following text has been added or revised:
- Line 345-355: "Previous studies have explored bioactive compounds from Spirulina with immunomodulatory effects; however, they did not specify individual compounds, instead examining Spirulina as whole extracts. While earlier work, including our own, employed network-based approaches to identify potential bioactive compounds from Spirulina, the present study provides novel mechanistic insight by specifically investigating the interaction between phycocyanobilin and LYN kinase. Unlike prior studies, we incorporated molecular docking, MD simulations, and binding free energy analyses, revealing the stability, binding mode, and key residue-level interactions of the phycocyanobilin–LYN complex. Furthermore, comparison with the native ligand strengthens the functional relevance of our findings. Collectively, these approaches provide detailed structural and mechanistic insight beyond association-based predictions."

---

2. The study focuses primarily on LYN after docking against several receptors (EGFR, FYN, HLA-B, LCK, LYN, and TP53). However, the criteria for selecting LYN as the primary target require further explanation. The authors should clarify why LYN was prioritized over FYN or LCK, which also showed favorable docking scores. Were statistical comparisons between docking scores performed?

Response:

- In the manuscript, we have explicitly stated that LYN was selected based on a combination of factors**,** not docking score alone. Although FYN and LCK also showed favorable docking energies, LYN was prioritized because: (i) it consistently emerged as a predicted target in both docking and independent network-based prediction (NetPredictor), (ii) it demonstrated strong and stable binding behavior in subsequent molecular dynamics simulations, and (iii) it has well-established relevance to SLE pathogenesis, particularly in regulating B-cell signaling and autoimmune responses. These clarifications have been added to improve transparency in target selection. [Line 153-156, Table 1; Line 165-175, Table 2; Line 204-213; 335-344]
- Regarding statistical comparison, we acknowledge that formal statistical testing of docking scores was not performed, as docking energies are primarily used for relative ranking rather than rigorous statistical inference.

----

3. The docking procedure requires additional methodological details to ensure reproducibility. The authors should specify the Grid box dimensions and coordinates. Number of docking runs. Exhaustiveness or search parameters used in AutoDock. Validation of the docking protocol (e.g., re-docking of native ligand). Without these details, it is difficult to assess the reliability of the docking results.

Response:

- As the above response, detailed docking parameters have been revised and added to the Materials and Methods and Results sections.
- Line 97-107: "To ensure comprehensive coverage of potential binding sites, the grid box was configured to encompass the relevant binding domains of each receptor, enabling thorough sampling of potential binding conformations. For the EGFR kinase domain, the grid box specifically included the W2R binding site and two SO4 sites. Detailed grid coordinates and exhaustiveness parameters were defined for each AutoDock run. The reliability of the docking protocol was validated by comparing the predicted binding energies of the studied compounds with those of native ligands from experimentally resolved crystal structures available in the PDB, using the same grid parameters and search settings. The accuracy of the protocol was confirmed by calculating the root-mean-square deviation (RMSD) between the predicted docking poses and the original crystal structures."
- Line 144-147: "The grid box was configured to encompass these binding sites, after which bilirubin and phycocyanobilin were docked to each receptor. The AutoDock results for both ligands, bilirubin and phycocyanobilin, against the selected receptors, as obtained using MGLTools, are summarized in Table 1."

- The docking protocol validation has been clarified, and the following text has been added or revised:
- Line 102-107: "The reliability of the docking protocol was validated by comparing the predicted binding energies of the studied compounds with those of native ligands from experimentally resolved crystal structures available in the PDB, using the same grid parameters and search settings. The accuracy of the protocol was confirmed by calculating the root-mean-square deviation (RMSD) between the predicted docking poses and the original crystal structures."

- Line 323-329: "To validate the docking protocol, the native ligand (staurosporine) was re-docked into the LYN kinase active site (PDB ID: 3A4O). The resulting RMSD value, maintained within ~2.5–3 Å, indicates that the docking procedure accurately reproduces the experimental binding mode, thereby supporting the reliability of the docking results. This structural consistency was further supported by MD simulations, in which the complex achieved equilibrium after 27 ns while maintaining a stable binding orientation."

----

4. Although MD simulations were conducted for 60 ns, several parameters are missing. The authors should report the temperature and pressure conditions used in simulations. Solvent model applied. Equilibration protocol. Time step used during simulations. Furthermore, a 60 ns simulation may be relatively short for stable protein–ligand interaction analysis. The authors should justify this duration.

Response:

- The MD simulations were conducted at a constant temperature of 300 K and a pressure of 1 atm, using the ff14SB force field. The system was prepared at pH 7.0 using PROPKA 3.0, and after energy minimization with the sander module, all bond and angle constraints were enforced using the SHAKE algorithm with a standard integration time step. Although the production run was 60 ns, the duration is justified as RMSD analysis confirmed the phycocyanobilin–LYN complex reached equilibrium after 27 ns. Furthermore, the complex and protein backbone maintained stable fluctuations of ~2.5–3 Å, while the Solvent Accessible Surface Area (SASA) remained stable at ~300 Å² after 20 ns. To ensure data reliability, the binding free energy (MM/PBSA and MM/GBSA) was calculated using only the last 10 ns of the stable, equilibrated trajectory, where phycocyanobilin demonstrated lower average fluctuations (RMSF) than the native ligand, staurosporine, indicating a highly stable complex. [Line 89-129]

----

5. The manuscript reports binding free energies using MM/PBSA and MM/GBSA methods. However, the difference between phycocyanobilin and the native ligand (staurosporine) should be statistically evaluated. Error bars or confidence intervals should be discussed more clearly. The biological relevance of these energy differences should be explained.

Response:

- The binding free energies (ΔG_bind) and non-covalent interaction energies for the phycocyanobilin–LYN and staurosporine–LYN complexes were calculated using the MM/PBSA and MM/GBSA methods, with results reported as mean values ± standard deviation to represent the energetic fluctuations over the stable production trajectory. For phycocyanobilin, the non-covalent interaction energy (-98.81 ± 5.84 kcal/mol) and ΔG_bind (-39.42 ± 8.41 kcal/mol) were significantly lower than those of the native inhibitor staurosporine (-77.33 ± 5.33 kcal/mol and -33.24 ± 5.57 kcal/mol, respectively), indicating a statistically stronger and more stable binding affinity. This energy difference is biologically significant as LYN kinase acts as a negative regulator of B-cell signaling; its loss or down-regulation is directly linked to the hyperactive immune responses seen in systemic lupus erythematosus (SLE). The superior stabilization of phycocyanobilin, particularly through key

residues like P21, T84, and M85, suggests its potential to effectively modulate LYN activity, offering a potential therapeutic mechanism similar to the protective role of its structural homolog, bilirubin, in reducing SLE disease activity. [Line 204-213; 221-231]

---

6. The conclusions suggest that phycocyanobilin could serve as a therapeutic compound for SLE. However, the study relies entirely on *in silico* approaches. The authors should clearly acknowledge this limitation. Avoid making strong therapeutic claims. Suggest future *in vitro* or in vivo validation experiments.

Response:

- We agree that our study is based on *in silico* approaches, which inherently have limitations. The manuscript has been revised to explicitly acknowledge this limitation in the Discussion section.
- Line 357-381: "However, this study is based entirely on *in silico* approaches, including molecular docking, target prediction, and MD simulations, which have inherent limitations in predicting biological activity in complex physiological systems. While replicate MD simulations were not included, the current simulation demonstrated stable and consistent protein–ligand interactions; additional independent runs would further support the robustness of the findings. To evaluate the suitability of phycocyanobilin as a therapeutic candidate for SLE, an *in silico* assessment was conducted using Lipinski's Rule of Five (RO5) [29], ADMET profiling, and related parameters. Phycocyanobilin has a molecular weight (MW) of 586.68 Da, lipophilicity (LogP) of 2.49, a topological polar surface area (TPSA) of 164.71 Å^2, ten rotatable bonds, five hydrogen bond donors, and seven hydrogen bond acceptors. It exceeds the RO5 threshold for molecular weight (>500 Da), representing a minor violation**;** however, its lipophilicity remains within the acceptable range (LogP < 5), supporting its potential for further optimization. ADMET profiling using ADMETlab 3.0 [30] indicates that, despite low predicted membrane permeability, phycocyanobilin demonstrates adequate human intestinal absorption and favorable oral bioavailability (F20%, F30%, and F50**%).** It also exhibits good natural product–likeness (NP score = 0.714), supporting its potential as a bioactive lead. Regarding safety, phycocyanobilin is derived from *A. platensis* (Spirulina), which has a well-established history of safe human consumption. Toxicity prediction using ProTox-3.0 [31] classifies phycocyanobilin as toxicity class 5, with a predicted LD_{50} of 3,066 mg/kg, indicating relatively low acute toxicity. Despite these limitations, its favorable safety profile, strong binding affinity to LYN kinase, and structural similarity to endogenous bilirubin support its potential as a lead compound for SLE drug development. Nevertheless, experimental validation is required. Future studies should include *in vitro* kinase assays and cell-based experiments (e.g., immune signaling or B-cell activation assays) to confirm its biological relevance in the context of SLE."

---

7. The discussion should better connect computational findings with biological mechanisms of SLE. Explain how LYN modulation affects immune signaling pathways. Discuss how phycocyanobilin may influence B-cell regulation and autoimmune responses. Include more recent literature on LYN signaling in SLE.

Response:

- As suggested by the reviewer, the Discussion has been revised to better connect the computational findings with the biological mechanisms of SLE. We expanded the text to explain the role of LYN as a key regulator of immune responses, B-cell differentiation, apoptosis, and cytokine signaling, and to describe how loss of LYN function is associated with lupus-like autoimmunity, hyperactive B cells, and myeloproliferation. We also clarified that the favorable binding of phycocyanobilin to the LYN ATP-binding pocket, along with its stable interactions with key residues, supports its potential to modulate LYN-associated signaling pathways relevant to B-cell regulation and autoimmune responses in SLE. In addition, the Discussion has been updated to include more recent literature on LYN signaling in SLE, including evidence linking LYN expression with disease activity, albuminuria, and renal pathology (Reference 28). [Line 290-381]

----

8. Several grammatical and stylistic issues should be corrected. Examples include:
   1. "MD simulations result demonstrates" → should be "MD simulation results demonstrate."
   2. "better docking interaction energy" → should be "more favorable docking interaction energy."
   3. Professional language editing is recommended.

Response:

- The manuscript has been revised for clarity and adherence to standard academic English. Grammatical and stylistic errors, including those noted, have been corrected, and sentence structure and terminology have been carefully edited throughout the text to improve readability, consistency, and overall presentation.

----

9. The abstract contains excessive numerical details. It would benefit from shortening technical descriptions. Highlighting the main biological implication.

Response:

- The abstract has been revised for clarity, with reduced technical detail and greater emphasis on the biological implications, particularly the role of phycocyanobilin in targeting LYN kinase in SLE.
- Line 26-37: "Phycocyanobilin, a bioactive compound derived from *Arthrospira platensis* C1, was investigated for its potential role in systemic lupus erythematosus (SLE) based on its structural similarity to bilirubin, with a Tanimoto score of 93%. Molecular docking revealed favorable binding affinities between phycocyanobilin and several protein targets, including EGFR, FYN, HLA-B, LCK, LYN, and TP53. Target prediction further identified LYN kinase as a key candidate. Molecular dynamics simulations demonstrated stable binding of the phycocyanobilin–LYN complex, with interaction profiles comparable to those of the native ligand, staurosporine. Binding free energy and residue-level analyses supported strong and stable interactions, highlighting key contributions to complex stability. Overall, these findings provide mechanistic insight into the interaction between phycocyanobilin and LYN, suggesting that this compound may modulate LYN-associated signaling pathways and warrants further investigation in the context of SLE."

---

10. Figures need improvement: Axis labels should be clearer in RMSD, RMSF, and SASA plots. Figure captions should describe the results more completely.

Response:

- The figures have been revised to improve clarity and presentation. Axis labels in the plots have been standardized and clarified. In addition, figure captions have been refined to more clearly describe the results and enhance interpretability. The figures have also been assessed using the NAAS tool (https://ngplosjournals.pagemajik.ai/artanalysis) recommended by the journal.

---

11. Several references contain formatting inconsistencies and duplicated entries (e.g., bilirubin signaling references). The reference list should be carefully checked in accordance with the journal's guidelines.

Response:

- We have carefully reviewed the reference list using Zotero and corrected formatting inconsistencies, removed duplicate entries, and ensured that all references comply with the journal's guidelines.
  - (The former Reference 4): The entry "Lamagna C, Hu Y, DeFranco AL, Lowell CA. B Cell–Specific Loss of Lyn Kinase Leads to Autoimmunity. The Journal of Immunology. 2014;192: 919–928. doi:10.4049/jimmunol.1301979" has been removed due to duplication, as it is already correctly listed as Reference 27.
  - (The former Reference 5): The entry "Vítek L. Bilirubin as a signaling molecule. Med Res Rev. 2020;40: 1335–1351. doi:10.1002/med.21660" has been removed due to duplication, as it is already correctly listed as Reference 7.
- Line 481-483: Reference 24 has been added: "Pettersen EF et al. UCSF Chimera--a visualization system for exploratory research and analysis. J Comput Chem 2004; 25(13): 1605–1612. doi:10.1002/jcc.20084."
- Line 498: Reference 29 has been added: "SwissADME. [cited 25 Apr 2026]. Available: https://www.swissadme.ch/"
- Line 499: Reference 30 has been added: "ADMETlab 3.0. [cited 25 Apr 2026]. Available: https://admetlab3.scbdd.com/"
- Line 500-501: Reference 31 has been added: "ProTox-3.0 - Prediction of TOXicity of chemicals. [cited 25 Apr 2026]. Available: https://tox.charite.de/protox3/index.php?site=compound_search_similarity"
