# Supplementary material for: Phycocyanobilin: A potential bioactive compound from Arthrospira platensis targeting LYN protein associated with systemic lupus erythematosus
Source: PLoS One. 2026 Aug 28;21(8):e0357093. doi: 10.1371/journal.pone.0357093 (PMC13524268; doi:10.1371/journal.pone.0357093)
Supplement: Supplementary file 4 [file pone.0357093.s008.pdf]

Response to Editor's and Reviewers' comments

PONE-D-25-50743R1

Response to Journal Requirements

1. If the reviewer comments include a recommendation to cite specific previously published works, please review and evaluate these publications to determine whether they are relevant and should be cited.

Response:
We carefully evaluated all references, and relevant publications have been incorporated into the revised manuscript where appropriate. References that were not directly relevant to the objectives, methodology, or findings of the present study were not included in the revised manuscript.

2. Please review your reference list to ensure that it is complete and correct. If you have cited papers that have been retracted, please include the rationale for doing so in the manuscript text, or remove these references and replace them with relevant current references. Any changes to the reference list should be mentioned in the rebuttal letter that accompanies your revised manuscript.

Response:
We thoroughly reviewed the reference list and verified the accuracy and completeness of all citations. We also checked for retracted publications and confirmed that none of the references cited in the revised manuscript have been retracted.

Response to Reviewer #1

We thank the reviewer for the thorough evaluation of our manuscript and for providing constructive comments. We have carefully considered all concerns and appreciate the insightful suggestions.

The objective of this study was to investigate the potential interaction between phycocyanobilin and LYN kinase through a focused *in silico* approach, including structural similarity analysis, molecular docking, molecular dynamics (MD) simulations, and binding free energy calculations. While several of the suggested analyses (e.g., PCA, DCCM, FEL, replicate MD simulations, and experimental

validation) would provide additional insights, they are beyond the scope of the current study.

1. The rationale for selecting EGFR, FYN, HLA-B, LCK, LYN, and TP53 as candidate targets is insufficiently justified and lacks a systematic target prioritization workflow.

Response:

The candidate targets were not selected arbitrarily. These proteins were initially identified through our previous report [Ref. 20], network-based analysis of systemic lupus erythematosus (SLE)-related targets associated with *Arthrospira platensis* (Spirulina) bioactive compounds. To evaluate the potential interactions of phycocyanobilin, all relevant receptor proteins identified in this analysis (EGFR, FYN, HLA-B, LCK, LYN, and TP53) were included in the initial molecular docking study. Additionally, to strengthen the target selection process we have performed KEGG pathway enrichment analysis in the revised manuscript:

Lines 95–98: "Docking simulations were conducted to evaluate the binding of bilirubin and phycocyanobilin to all potential receptors, including EGFR (binding site 1: W2R; binding sites 2 and 3: SO4), FYN, HLA-B, LCK, LYN, and TP53, which were initially identified through our previous report [20]."

Lines 161–168: "To further strengthen the target selection process, we performed KEGG pathway enrichment analysis. Pathways were mapped using the KEGG database, and enrichment was assessed using a hypergeometric test. The selected proteins were significantly enriched in immune-related pathways, including T-cell receptor signaling, NF-kappa B signaling, Fc epsilon RI signaling, and natural killer cell-mediated cytotoxicity (Figures in S3 Fig). These findings further support the biological relevance of the selected targets in immune regulation and SLE pathogenesis."

At this stage, the study was not specifically focused on LYN; instead, molecular docking was systematically performed across all candidate targets. Target prioritization was subsequently based on the integration of molecular docking and target prediction results. Among the evaluated proteins, LYN consistently emerged as one of the highest-ranked targets. In addition, NetPredictor independently identified LYN as a potential molecular target of phycocyanobilin, while molecular docking demonstrated favorable binding affinities (approximately −9 to −10 kcal/mol; Supplementary Table S1). Collectively, the convergent evidence from network pharmacology, KEGG pathway enrichment

analysis, target prediction, and molecular docking provides a systematic rationale for prioritizing LYN for subsequent molecular dynamics simulations and detailed mechanistic investigation.

Lines 527–530: "S3 Fig. KEGG pathway enrichment analysis of the selected protein targets (EGFR, FYN, HLA-B, LCK, LYN, and TP53). Bubble plot showing significantly enriched KEGG pathways for the selected protein targets. Bubble size represents the number of associated genes, and color indicates the enrichment *Q* value."

2. No transcriptomic, proteomic, GWAS, or pathway-enrichment analysis was performed to support target selection in SLE.

Response:

We appreciate this valuable suggestion. Although transcriptomic, proteomic, and GWAS data were not incorporated into the present study, we incorporated KEGG pathway enrichment analysis to provide systems-level support for target selection. This analysis demonstrated significant enrichment of immune-related pathways associated with SLE pathogenesis, including T-cell receptor signaling, NF-kappa B signaling, Fc epsilon RI signaling, and natural killer cell-mediated cytotoxicity. [**Lines 161–168, 527–530**]

3. The study assumes that binding to LYN kinase may be therapeutically beneficial without demonstrating whether phycocyanobilin acts as an inhibitor, activator, or modulator.
4. The biological implications of LYN modulation in SLE are oversimplified despite the known dual regulatory role of LYN in immune signaling.

Response 3–4:

We have revised the manuscript to avoid overinterpretation and stated that phycocyanobilin may modulate LYN-mediated signaling pathways. We have also expanded the Discussion to acknowledge the context-dependent dual regulatory role of LYN in immune signaling.

Lines 316–318: "Consequently, the favorable binding affinity of phycocyanobilin toward LYN suggests that it may interact with LYN and potentially modulate LYN-mediated signaling pathways."

Lines 391–395: "The function of LYN in immune regulation is context-dependent. Although phycocyanobilin exhibited stable binding to the LYN kinase domain in the present study, future studies should include kinase selectivity profiling, *in vitro* kinase assays, and cell-based experiments (e.g., immune

signaling and B-cell activation assays) to confirm its biological relevance and target specificity of phycocyanobilin in SLE."

In the Conclusions (Lines 399–404): "Multiple complementary computational approaches, including molecular docking, target prediction, pathway enrichment, molecular dynamics simulations, and binding free energy analyses, consistently identified LYN as a biologically relevant target and demonstrated stable binding of phycocyanobilin to LYN. Collectively, these findings suggest that phycocyanobilin warrants further investigation as a potential modulator of LYN-associated signaling pathways in SLE."

5. Docking validation is insufficiently reported; re-docking RMSD values and validation metrics are not presented.

Response:

Docking validation was performed by re-docking the native ligand, staurosporine, into the LYN active site. The resulting RMSD demonstrated acceptable agreement with the experimental binding mode, supporting the reliability of the docking protocol. This information has already been explicitly included in the revised manuscript.

Lines 334–340: "To validate the docking protocol, the native ligand (staurosporine) was re-docked into the LYN kinase active site (PDB ID: 3A4O). The resulting RMSD value, maintained within ~2.5–3 Å, indicates that the docking procedure accurately reproduces the experimental binding mode, thereby supporting the reliability of the docking results. This structural consistency was further supported by MD simulations, in which the complex achieved equilibrium after 27 ns while maintaining a stable binding orientation."

6. The conclusion that phycocyanobilin binds more strongly than staurosporine is not fully supported because the MM-GBSA energy difference falls within the reported error range.

Response:

As suggested by the reviewer, the changes have been made.

Lines 216–223: "Calculations using the MM/PBSA and MM/GBSA methods yielded consistent estimates of binding free energy. Electrostatic and vdW interactions were the major contributors to the non-covalent binding energy. As shown in Table 3, the non-covalent interaction energy of phycocyanobilin (−98.81 ± 5.84 kcal/mol) was lower than that of the native ligand, staurosporine (−77.33 ± 5.33 kcal/mol), suggesting a trend toward more favorable binding of phycocyanobilin to LYN. The calculated binding free energy (ΔG_bind) of

phycocyanobilin (−39.42 ± 8.41 kcal/mol) was likewise more favorable than that of staurosporine (−33.24 ± 5.57 kcal/mol)."

This interpretation is further supported by multiple complementary computational analyses, including molecular dynamics (MD) stability, per-residue energy decomposition, and hydrogen-bond interaction analyses.

7. Molecular dynamics simulations were limited to a single 60 ns trajectory, which may not adequately capture long-term conformational stability.
8. No independent replicate MD simulations were performed to assess reproducibility and convergence.

Response 7–8:

We performed a 60-ns molecular dynamics (MD) simulation, which is consistent with simulation durations commonly employed in protein–ligand interaction studies to evaluate structural stability and binding behavior. In the present study, the RMSD trajectories reached a stable plateau, indicating that the complexes had reached equilibrium and supporting the reliability of the subsequent trajectory-based analyses.

9. No principal component analysis (PCA) was conducted to evaluate dominant protein motions during simulation.
10. Dynamic cross-correlation analysis was not performed to investigate communication between protein regions upon ligand binding.
11. Free-energy landscape (FEL) analysis was not included to identify stable conformational states of the protein–ligand complex.

Response 9–11:

We agree that these analyses may provide additional insights into conformational dynamics and correlated motions. However, they were beyond the scope of the present study, which focused primarily on evaluating binding stability and protein–ligand interaction patterns.

12. Ensemble docking was not performed, potentially overlooking receptor flexibility and alternative binding conformations.
13. Docking results rely on a single docking engine (AutoDock) without cross-validation using additional docking platforms.

Response 12–13:

The use of multiple docking approaches can improve the robustness of docking predictions. In addition to AutoDock, we performed molecular docking using

CDOCKER, FlexX, and iGEMDOCK. The results from these independent docking methods were consistent with those obtained using AutoDock, with LYN consistently ranking among the highest-scoring targets for phycocyanobilin across all evaluated receptors. AutoDock is also a widely used and well-validated platform for protein–ligand docking studies. Furthermore, the docking findings were supported by molecular dynamics simulations and MM/GBSA binding free energy analyses, which consistently demonstrated stable binding of phycocyanobilin within the LYN active site.

Lines 148−152: "In addition to AutoDock, we performed molecular docking using CDOCKER, FlexX, and iGEMDOCK. The results from these independent docking methods were consistent with those obtained using AutoDock, with LYN consistently ranking among the top-scoring targets for phycocyanobilin across all evaluated receptors. (Figure in S2 Fig)."

Lines 523−526: "S2 Fig. Comparison of molecular docking results of phycocyanobilin with selected protein receptors using AutoDock, FlexX, and iGEMDOCK. Docking scores were generated using the native scoring function implemented in each docking program and therefore should not be directly compared numerically across different software platforms."

14. No kinase selectivity assessment was performed to determine whether phycocyanobilin preferentially targets LYN over related kinases.

Response:

We agree that kinase selectivity is an important consideration for future drug development. However, the objective of the present study was to identify and characterize a potential interaction between phycocyanobilin and LYN rather than establish its kinase selectivity profile. Kinase selectivity cannot be reliably inferred from molecular docking alone, as comprehensive selectivity profiling typically requires experimental kinase panel screening, such as enzymatic inhibition assays or kinome-wide profiling. These evaluations are generally performed during subsequent stages of drug development rather than in an initial in silico screening study. Future studies incorporating experimental kinase panel assays will be necessary to determine whether phycocyanobilin exhibits selectivity for LYN over other Src family kinases and additional members of the kinome.

15. The study lacks comparison with clinically relevant SLE therapeutics or known LYN-targeting compounds.

Response:

The primary objective of this study was to identify phycocyanobilin as a potential LYN-binding compound through an *in silico* drug discovery approach rather than to compare its binding properties with clinically approved therapies for SLE. Most currently approved SLE therapeutics, including corticosteroids, antimalarials, immunosuppressants, and biologics, do not directly target LYN kinase, making direct comparisons at the molecular docking level difficult. Instead, we compared the binding of phycocyanobilin with that of the co-crystallized LYN inhibitor, staurosporine, which served as a reference ligand for docking validation and molecular dynamics simulations. We agree that future studies should include comparisons with selective LYN or Src family kinase inhibitors and evaluate phycocyanobilin alongside clinically relevant SLE therapeutics in biochemical and cellular models to further define its therapeutic potential.

16. ADMET analysis indicates limited drug-likeness characteristics (high molecular weight and high TPSA), yet these limitations are not critically discussed.

Response:

This limitation was partially discussed in the previous revised manuscript. In this revised manuscript, however, we have expanded the Discussion to critically discuss the implications of the ADMET predictions. Specifically, we now note that although phycocyanobilin exhibits one violation of Lipinski's Rule of Five (molecular weight >500 Da) and a relatively high topological polar surface area (TPSA), these characteristics are not uncommon among naturally occurring bioactive compounds and may contribute to reduced passive membrane permeability and oral absorption. We have added the following statement to the Discussion:

Lines 372–378: "Its relatively high MW and TPSA suggest that passive membrane permeability and oral absorption may be lower than those of compounds that fully satisfy Lipinski's RO5. Nevertheless, these properties do not preclude its potential as a lead compound, as many natural products possess physicochemical characteristics outside conventional drug-likeness criteria while retaining biological activity. Further lead optimization or formulation strategies may improve its pharmacokinetic properties."

17. The relationship between structural similarity to bilirubin and functional similarity is assumed rather than experimentally demonstrated.

Response:

We agree that structural similarity does not guarantee functional similarity. In the present study, phycocyanobilin showed a high Tanimoto similarity score (93%) to bilirubin, indicating a very close structural relationship within the bile pigment chemical space. Accordingly, this similarity was used only as a rationale for further investigation, supporting the selection of phycocyanobilin for downstream computational analysis rather than serving as evidence of functional equivalence. We have revised the manuscript to clarify that structural similarity suggests a potential relationship in chemical space, while biological activity must be confirmed through experimental validation.

Lines 306–311: "Structural similarity analysis revealed a high Tanimoto score of 93% between phycocyanobilin and bilirubin. Both compounds share a high degree of structural similarity as tetrapyrrole chromophores. According to PubChem, a Tanimoto score of ≥90% indicates a high level of confidence, suggesting that their core structures and key functional groups are sufficiently similar and occupy closely related chemical space. However, structural similarity alone does not predict equivalent biological activity, which requires experimental validation."

18. The predicted target list generated by NetPredictor was not further validated through network pharmacology or biological pathway analysis.

Response:

The target prediction analysis using NetPredictor was employed as an initial computational step to expand the potential protein target space for Spirulina-derived compounds. NetPredictor generates predicted drug–target interactions based on three input matrices: (i) drug–target binary associations, (ii) target similarity, and (iii) drug similarity, thereby providing a data-driven prediction of possible interactions. To improve robustness, we further prioritized candidate genes using AutoHGPEC (a Cytoscape plugin), which integrates gene ranking with sequence similarity information derived from BLASTP analysis. This complementary approach was used to refine and prioritize biologically relevant targets. Importantly, the final intersecting target between NetPredictor predictions and AutoHGPEC prioritization was LYN kinase, which was selected as the primary target for downstream molecular docking and simulation analyses.

19. Residue decomposition and hydrogen-bond analyses are descriptive and lack statistical evaluation of interaction persistence.

Response:

Residue decomposition and hydrogen-bond occupancy analyses were performed descriptively to identify key stabilizing interactions within the complex. We agree that additional statistical analyses would provide further quantitative insight into these interaction patterns. Regarding our limitation, we have modified the wording as response in the comment 6, for example.

20. The manuscript remains entirely computational and does not provide experimental validation (e.g., kinase assays, cellular assays, or mechanistic studies) to support the proposed therapeutic relevance in SLE.

Response:

We have already addressed this concern in our previous response. As noted, experimental validation—including kinase activity assays, cell-based experiments, and ultimately in vivo studies—is essential to establish the biological relevance of phycocyanobilin in SLE. We have explicitly acknowledged this limitation in the revised manuscript and emphasized that the present work is a hypothesis-generating computational study that provides a structural and mechanistic foundation for future experimental validation. [**Lines 390–395**]

Response to Reviewer #2

We are highly grateful to the reviewer for the insightful comments on our manuscript and recommending its acceptance for publication.
