# Supplementary material for: Phycocyanobilin: A potential bioactive compound from Arthrospira platensis targeting LYN protein associated with systemic lupus erythematosus
Source: PLoS One. 2026 Aug 28;21(8):e0357093. doi: 10.1371/journal.pone.0357093 (PMC13524268; doi:10.1371/journal.pone.0357093)
Supplement: Peer review history

# Peer review: Decision Letter 0

**DOI:** 10.1371/journal.pone.0357093.r001

**Authors:** Eman Zahran

19 Mar 2026

Dear Dr. Ruengjitchatchawalya,

Thank you for submitting your manuscript to PLOS ONE. After careful consideration, we feel that it has merit but does not fully meet PLOS ONE’s publication criteria as it currently stands. Therefore, we invite you to submit a revised version of the manuscript that addresses the points raised during the review process.

==============================

Dear Authors,

The reviewers have raised several substantial concerns that must be carefully and comprehensively addressed before the manuscript can be reconsidered. Detailed reviewer comments are provided below to guide you in revising the manuscript. Upon submitting a revised version, please ensure that all reviewer comments are addressed point by point in a separate response document.

==============================

Please submit your revised manuscript by May 03 2026 11:59PM. If you will need more time than this to complete your revisions, please reply to this message or contact the journal office at plosone@plos.org. When you're ready to submit your revision, log on to https://www.editorialmanager.com/pone/ and select the 'Submissions Needing Revision' folder to locate your manuscript file.

- A letter that responds to each point raised by the academic editor and reviewer(s). You should upload this letter as a separate file labeled 'Response to Reviewers'.
- A marked-up copy of your manuscript that highlights changes made to the original version. You should upload this as a separate file labeled 'Revised Manuscript with Track Changes'.
- An unmarked version of your revised paper without tracked changes. You should upload this as a separate file labeled 'Manuscript'.

If applicable, we recommend that you deposit your laboratory protocols in protocols.io to enhance the reproducibility of your results. Protocols.io assigns your protocol its own identifier (DOI) so that it can be cited independently in the future. For instructions see: https://journals.plos.org/plosone/s/submission-guidelines#loc-laboratory-protocols. Additionally, PLOS ONE offers an option for publishing peer-reviewed Lab Protocol articles, which describe protocols hosted on protocols.io. Read more information on sharing protocols at https://plos.org/protocols?utm_medium=editorial-email&utm_source=authorletters&utm_campaign=protocols.

We look forward to receiving your revised manuscript.

Kind regards,

Eman Zahran, Ph.D

Academic Editor

PLOS One

Journal Requirements:

When submitting your revision, we need you to address these additional requirements.

1. Please ensure that your manuscript meets PLOS ONE's style requirements, including those for file naming. The PLOS ONE style templates can be found at

https://journals.plos.org/plosone/s/file?id=wjVg/PLOSOne_formatting_sample_main_body.pdf and https://journals.plos.org/plosone/s/file?id=ba62/PLOSOne_formatting_sample_title_authors_affiliations.pdf

2. Please note that PLOS One has specific guidelines on code sharing for submissions in which author-generated code underpins the findings in the manuscript. In these cases, we expect all author-generated code to be made available without restrictions upon publication of the work. Please review our guidelines at https://journals.plos.org/plosone/s/materials-and-software-sharing#loc-sharing-code and ensure that your code is shared in a way that follows best practice and facilitates reproducibility and reuse.

3. Please amend either the title on the online submission form (via Edit Submission) or the title in the manuscript so that they are identical.

4. We note that Figure 4, S2, S3, in your submission contain copyrighted images. All PLOS content is published under the Creative Commons Attribution License (CC BY 4.0), which means that the manuscript, images, and Supporting Information files will be freely available online, and any third party is permitted to access, download, copy, distribute, and use these materials in any way, even commercially, with proper attribution. For more information, see our copyright guidelines: http://journals.plos.org/plosone/s/licenses-and-copyright.

We require you to either (1) present written permission from the copyright holder to publish these figures specifically under the CC BY 4.0 license, or (2) remove the figures from your submission:

a. You may seek permission from the original copyright holder of Figure 4, S2, S3, to publish the content specifically under the CC BY 4.0 license.

We recommend that you contact the original copyright holder with the Content Permission Form (http://journals.plos.org/plosone/s/file?id=7c09/content-permission-form.pdf) and the following text:

“I request permission for the open-access journal PLOS ONE to publish XXX under the Creative Commons Attribution License (CCAL) CC BY 4.0 (http://creativecommons.org/licenses/by/4.0/). Please be aware that this license allows unrestricted use and distribution, even commercially, by third parties. Please reply and provide explicit written permission to publish XXX under a CC BY license and complete the attached form.”

Please upload the completed Content Permission Form or other proof of granted permissions as an "Other" file with your submission.

In the figure caption of the copyrighted figure, please include the following text: “Reprinted from [ref] under a CC BY license, with permission from [name of publisher], original copyright [original copyright year].”

b. If you are unable to obtain permission from the original copyright holder to publish these figures under the CC BY 4.0 license or if the copyright holder’s requirements are incompatible with the CC BY 4.0 license, please either i) remove the figure or ii) supply a replacement figure that complies with the CC BY 4.0 license. Please check copyright information on all replacement figures and update the figure caption with source information. If applicable, please specify in the figure caption text when a figure is similar but not identical to the original image and is therefore for illustrative purposes only.

5. If the reviewer comments include a recommendation to cite specific previously published works, please review and evaluate these publications to determine whether they are relevant and should be cited. There is no requirement to cite these works unless the editor has indicated otherwise.

6. Please review your reference list to ensure that it is complete and correct. If you have cited papers that have been retracted, please include the rationale for doing so in the manuscript text, or remove these references and replace them with relevant current references. Any changes to the reference list should be mentioned in the rebuttal letter that accompanies your revised manuscript. If you need to cite a retracted article, indicate the article’s retracted status in the References list and also include a citation and full reference for the retraction notice.

[Note: HTML markup is below. Please do not edit.]

Reviewers' comments:

Reviewer's Responses to Questions

**Comments to the Author**

1. Is the manuscript technically sound, and do the data support the conclusions?

Reviewer #1: Yes

Reviewer #2: Yes

**********

2. Has the statistical analysis been performed appropriately and rigorously?

Reviewer #1: Yes

Reviewer #2: Yes

**********

3. Have the authors made all data underlying the findings in their manuscript fully available?

The PLOS Data policy

Reviewer #1: Yes

Reviewer #2: Yes

**********

4. Is the manuscript presented in an intelligible fashion and written in standard English?

Reviewer #1: Yes

Reviewer #2: No

**********

Reviewer #1: Thank you for presenting an interesting and promising research idea. The concept of exploring phycocyanobilin from Arthrospira platensis as a potential modulator of the LYN protein associated with systemic lupus erythematosus is valuable and relevant. This work contributes to the growing field of computational drug discovery and may provide a basis for future experimental investigations.

1 The study relies solely on in-silico predictions Authors should clearly acknowledge this limitation and suggest experimental validation (in vitro kinase assay or cell-based validation).

2 Docking protocol validation is not clearly described Provide re-docking of the native ligand and report RMSD values to validate the docking protocol.

3 Lack of detailed docking parameters Include grid box dimensions, scoring function, exhaustiveness, and docking software settings.

4 Limited comparison with known inhibitors of LYN Compare phycocyanobilin with established LYN kinase inhibitors to contextualize binding affinity.

5 Binding site analysis is insufficient Provide detailed analysis of key amino acid residues involved in binding, hydrogen bonds, hydrophobic interactions, and π-interactions.

6 Protein structure preparation not fully described Clarify how the LYN protein structure was prepared (removal of water molecules, protonation states, energy minimization).

7 MD simulation duration may be insufficient Extend MD simulations or justify simulation length to ensure system stability and reliability.

8 Trajectory analysis is limited Include additional MD parameters such as RMSF, radius of gyration (Rg), SASA, and hydrogen bond analysis.

9 Energy decomposition analysis is missing Perform per-residue energy decomposition to identify residues contributing most to ligand binding.

10 No ADMET or drug-likeness evaluation Evaluate pharmacokinetic properties such as Lipinski rules, ADMET, toxicity prediction, and bioavailability.

11 Lack of pathway or biological context discussion Discuss how inhibition of LYN may mechanistically influence SLE-related signaling pathways.

12 Network pharmacology analysis could strengthen the study Include protein–protein interaction or pathway enrichment analysis to support biological relevance.

13 Structural similarity to bilirubin is mentioned but not deeply analyzed Provide a structure-activity comparison and potential functional implications of this similarity.

14 Figures may lack clarity in interaction visualization Improve structural visualization showing 2D and 3D interaction maps of ligand–protein complexes.

15 Statistical robustness of simulation results is unclear Include replicate simulations or statistical confidence intervals for binding energy calculations.

Reviewer #2: Major Comments

• While the manuscript highlights the potential interaction between phycocyanobilin and LYN kinase, the novelty should be clarified more explicitly. Previous studies have already explored bioactive compounds from Spirulina with immunomodulatory effects. The authors should clearly state: What new mechanistic insight does this study provide? How do their findings differ from the previously published network analysis study referenced in the manuscript?

• The study focuses primarily on LYN after docking against several receptors (EGFR, FYN, HLA-B, LCK, LYN, and TP53). However, the criteria for selecting LYN as the primary target require further explanation. The authors should clarify why LYN was prioritized over FYN or LCK, which also showed favorable docking scores. Were statistical comparisons between docking scores performed?

• The docking procedure requires additional methodological details to ensure reproducibility. The authors should specify the Grid box dimensions and coordinates. Number of docking runs. Exhaustiveness or search parameters used in AutoDock. Validation of the docking protocol (e.g., re-docking of native ligand). Without these details, it is difficult to assess the reliability of the docking results.

• Although MD simulations were conducted for 60 ns, several parameters are missing. The authors should report the temperature and pressure conditions used in simulations. Solvent model applied. Equilibration protocol. Time step used during simulations. Furthermore, a 60 ns simulation may be relatively short for stable protein–ligand interaction analysis. The authors should justify this duration.

• The manuscript reports binding free energies using MM/PBSA and MM/GBSA methods. However, the difference between phycocyanobilin and the native ligand (staurosporine) should be statistically evaluated. Error bars or confidence intervals should be discussed more clearly. The biological relevance of these energy differences should be explained.

• The conclusions suggest that phycocyanobilin could serve as a therapeutic compound for SLE. However, the study relies entirely on in silico approaches. The authors should clearly acknowledge this limitation. Avoid making strong therapeutic claims. Suggest future in vitro or in vivo validation experiments.

• The discussion should better connect computational findings with biological mechanisms of SLE. Explain how LYN modulation affects immune signaling pathways. Discuss how phycocyanobilin may influence B-cell regulation and autoimmune responses. Include more recent literature on LYN signaling in SLE.

• Several grammatical and stylistic issues should be corrected. Examples include:

1. “MD simulations result demonstrates” → should be “MD simulation results demonstrate.”

2. “better docking interaction energy” → should be “more favorable docking interaction energy.”

3. Professional language editing is recommended.

• The abstract contains excessive numerical details. It would benefit from shortening technical descriptions. Highlighting the main biological implication.

• Figures need improvement: Axis labels should be clearer in RMSD, RMSF, and SASA plots. Figure captions should describe the results more completely.

• Several references contain formatting inconsistencies and duplicated entries (e.g., bilirubin signaling references). The reference list should be carefully checked in accordance with the journal's guidelines.

**********

what does this mean?). If published, this will include your full peer review and any attached files.

If you choose “no”, your identity will remain anonymous but your review may still be made public.

**Do you want your identity to be public for this peer review?**  For information about this choice, including consent withdrawal, please see our Privacy Policy

Reviewer #1: **Yes:**  Omar A. Ahmed-farid

Reviewer #2: No

**********

[NOTE: If reviewer comments were submitted as an attachment file, they will be attached to this email and accessible via the submission site. Please log into your account, locate the manuscript record, and check for the action link "View Attachments". If this link does not appear, there are no attachment files.]

To ensure your figures meet our technical requirements, please review our figure guidelines: https://journals.plos.org/plosone/s/figures

You may also use PLOS’s free figure tool, NAAS, to help you prepare publication quality figures: https://journals.plos.org/plosone/s/figures#loc-tools-for-figure-preparation.

NAAS will assess whether your figures meet our technical requirements by comparing each figure against our figure specifications.

Attachment Submitted filename: PONE-D-25-50743_ Comments.pdf

# Author comment: Author response to Decision Letter 1

**DOI:** 10.1371/journal.pone.0357093.r002

6 May 2026

Response to Reviewers file has been attached.

Attachment Submitted filename: Response to Reviewers_Chaiprasert et al (30-4-2026).pdf

# Peer review: Decision Letter 1

**DOI:** 10.1371/journal.pone.0357093.r003

**Authors:** Eman Zahran

9 Jun 2026

Dear Dr. Ruengjitchatchawalya,

Thank you for submitting your manuscript to PLOS ONE. After careful consideration, we feel that it has merit but does not fully meet PLOS ONE’s publication criteria as it currently stands. Therefore, we invite you to submit a revised version of the manuscript that addresses the points raised during the review process.

==============================

Dear Authors,

Thank you for submitting your manuscript. The manuscript has now been evaluated by external peer reviewers and assessed at the editorial level.

The reviewers have raised minor revisions that must be carefully and comprehensively addressed before the manuscript can be reconsidered. Detailed reviewer comments are provided below to guide you in revising the manuscript.

upon submitting a revised version, please ensure that all reviewer comments are addressed point-by-point in a separate response document.

Thank you for considering the journal for your work. We look forward to receiving your revised submission.

==============================

Please submit your revised manuscript by Jul 24 2026 11:59PM. If you will need more time than this to complete your revisions, please reply to this message or contact the journal office at plosone@plos.org. When you're ready to submit your revision, log on to https://www.editorialmanager.com/pone/ and select the 'Submissions Needing Revision' folder to locate your manuscript file.

- A letter that responds to each point raised by the academic editor and reviewer(s). You should upload this letter as a separate file labeled 'Response to Reviewers'.
- A marked-up copy of your manuscript that highlights changes made to the original version. You should upload this as a separate file labeled 'Revised Manuscript with Track Changes'.
- An unmarked version of your revised paper without tracked changes. You should upload this as a separate file labeled 'Manuscript'.

If you would like to make changes to your financial disclosure, please include your updated statement in your cover letter. Guidelines for resubmitting your figure files are available below the reviewer comments at the end of this letter.

If applicable, we recommend that you deposit your laboratory protocols in protocols.io to enhance the reproducibility of your results. Protocols.io assigns your protocol its own identifier (DOI) so that it can be cited independently in the future. For instructions see: https://journals.plos.org/plosone/s/submission-guidelines#loc-laboratory-protocols. Additionally, PLOS ONE offers an option for publishing peer-reviewed Lab Protocol articles, which describe protocols hosted on protocols.io. Read more information on sharing protocols at https://plos.org/protocols?utm_medium=editorial-email&utm_source=authorletters&utm_campaign=protocols.

As the corresponding author, your ORCID iD is verified in the submission system and will appear in the published article. PLOS supports the use of ORCID, and we encourage all coauthors to register for an ORCID iD and use it as well. Please encourage your coauthors to verify their ORCID iD within the submission system before final acceptance, as unverified ORCID iDs will not appear in the published article. *Only*  the individual author can complete the verification step; PLOS staff *cannot*  verify ORCID iDs on behalf of authors.

We look forward to receiving your revised manuscript.

Kind regards,

Eman Zahran, Ph.D

Academic Editor

PLOS One

**Journal Requirements:**

1. If the reviewer comments include a recommendation to cite specific previously published works, please review and evaluate these publications to determine whether they are relevant and should be cited. There is no requirement to cite these works unless the editor has indicated otherwise.

2. Please review your reference list to ensure that it is complete and correct. If you have cited papers that have been retracted, please include the rationale for doing so in the manuscript text, or remove these references and replace them with relevant current references. Any changes to the reference list should be mentioned in the rebuttal letter that accompanies your revised manuscript. If you need to cite a retracted article, indicate the article’s retracted status in the References list and also include a citation and full reference for the retraction notice.

[Note: HTML markup is below. Please do not edit.]

Reviewers' comments:

Reviewer's Responses to Questions

**Comments to the Author**

Reviewer #1: All comments have been addressed

Reviewer #2: All comments have been addressed

**********

2. Is the manuscript technically sound, and do the data support the conclusions?

Reviewer #1: Yes

Reviewer #2: Yes

**********

3. Has the statistical analysis been performed appropriately and rigorously?

Reviewer #1: No

Reviewer #2: Yes

**********

4. Have the authors made all data underlying the findings in their manuscript fully available?

The PLOS Data policy

Reviewer #1: No

Reviewer #2: Yes

**********

5. Is the manuscript presented in an intelligible fashion and written in standard English?

Reviewer #1: No

Reviewer #2: Yes

**********

Reviewer #1: 1 The rationale for selecting EGFR, FYN, HLA-B, LCK, LYN, and TP53 as candidate targets is insufficiently justified and lacks a systematic target prioritization workflow.

2 No transcriptomic, proteomic, GWAS, or pathway-enrichment analysis was performed to support target selection in SLE.

3 The study assumes that binding to LYN kinase may be therapeutically beneficial without demonstrating whether phycocyanobilin acts as an inhibitor, activator, or modulator.

4 The biological implications of LYN modulation in SLE are oversimplified despite the known dual regulatory role of LYN in immune signaling.

5 Docking validation is insufficiently reported; re-docking RMSD values and validation metrics are not presented.

6 The conclusion that phycocyanobilin binds more strongly than staurosporine is not fully supported because the MM-GBSA energy difference falls within the reported error range.

7 Molecular dynamics simulations were limited to a single 60 ns trajectory, which may not adequately capture long-term conformational stability.

8 No independent replicate MD simulations were performed to assess reproducibility and convergence.

9 No principal component analysis (PCA) was conducted to evaluate dominant protein motions during simulation.

10 Dynamic cross-correlation analysis was not performed to investigate communication between protein regions upon ligand binding.

11 Free-energy landscape (FEL) analysis was not included to identify stable conformational states of the protein–ligand complex.

12 Ensemble docking was not performed, potentially overlooking receptor flexibility and alternative binding conformations.

13 Docking results rely on a single docking engine (AutoDock) without cross-validation using additional docking platforms.

14 No kinase selectivity assessment was performed to determine whether phycocyanobilin preferentially targets LYN over related kinases.

15 The study lacks comparison with clinically relevant SLE therapeutics or known LYN-targeting compounds.

16 ADMET analysis indicates limited drug-likeness characteristics (high molecular weight and high TPSA), yet these limitations are not critically discussed.

17 The relationship between structural similarity to bilirubin and functional similarity is assumed rather than experimentally demonstrated.

18 The predicted target list generated by NetPredictor was not further validated through network pharmacology or biological pathway analysis.

19 Residue decomposition and hydrogen-bond analyses are descriptive and lack statistical evaluation of interaction persistence.

20 The manuscript remains entirely computational and does not provide experimental validation (e.g., kinase assays, cellular assays, or mechanistic studies) to support the proposed therapeutic relevance in SLE.

Reviewer #2: I have carefully reviewed the revised version of the paper "Phycocyanobilin: A potential bioactive compound from Arthrospira platensis targeting LYN protein associated with systemic lupus erythematosus" by the author and thoroughly checked the responses to the modification suggestions I put forward earlier. The author not only provided a detailed explanation of each modification item but also made substantial improvements in the main text. These revisions have significantly enhanced the paper's academic rigor and readability, fully demonstrating the author's emphasis on academic norms and a serious attitude towards the review comments. I recommend its acceptance and publication.

**********

what does this mean?). If published, this will include your full peer review and any attached files.

If you choose “no”, your identity will remain anonymous but your review may still be made public.

**Do you want your identity to be public for this peer review?**  For information about this choice, including consent withdrawal, please see our Privacy Policy

Reviewer #1: No

Reviewer #2: No

**********

[NOTE: If reviewer comments were submitted as an attachment file, they will be attached to this email and accessible via the submission site. Please log into your account, locate the manuscript record, and check for the action link "View Attachments". If this link does not appear, there are no attachment files.]

To ensure your figures meet our technical requirements, please review our figure guidelines: https://journals.plos.org/plosone/s/figures

You may also use PLOS’s free figure tool, NAAS, to help you prepare publication quality figures: https://journals.plos.org/plosone/s/figures#loc-tools-for-figure-preparation.

NAAS will assess whether your figures meet our technical requirements by comparing each figure against our figure specifications.

# Author comment: Author response to Decision Letter 2

**DOI:** 10.1371/journal.pone.0357093.r004

24 Jul 2026

Response to reviewers has been attached.

Attachment Submitted filename: Response to Reviewers_PONE-D-25-50743R1.pdf

# Peer review: Decision Letter 2

**DOI:** 10.1371/journal.pone.0357093.r005

**Authors:** Eman Zahran

12 Aug 2026

Phycocyanobilin: A potential bioactive compound from Arthrospira platensis targeting LYN protein associated with systemic lupus erythematosus

PONE-D-25-50743R2

Dear Dr. Ruengjitchatchawalya,

We’re pleased to inform you that your manuscript has been judged scientifically suitable for publication and will be formally accepted for publication once it meets all outstanding technical requirements.

Within one week, you’ll receive an e-mail detailing the required amendments. When these have been addressed, you’ll receive a formal acceptance letter and your manuscript will be scheduled for publication.

An invoice will be generated when your article is formally accepted. Please note, if your institution has a publishing partnership with PLOS and your article meets the relevant criteria, all or part of your publication costs will be covered. Please make sure your user information is up-to-date by logging into Editorial Manager at Editorial Manager® and clicking the ‘Update My Information' link at the top of the page. For questions related to billing, please contact billing support.

If your institution or institutions have a press office, please notify them about your upcoming paper to help maximize its impact. If they’ll be preparing press materials, please inform our press team as soon as possible -- no later than 48 hours after receiving the formal acceptance. Your manuscript will remain under strict press embargo until 2 pm Eastern Time on the date of publication. For more information, please contact onepress@plos.org.

Kind regards,

Eman Zahran, Ph.D

Academic Editor

PLOS One

Additional Editor Comments (optional):

Reviewers' comments:

Reviewer's Responses to Questions

**Comments to the Author**

Reviewer #1: All comments have been addressed

**********

2. Is the manuscript technically sound, and do the data support the conclusions?

Reviewer #1: Yes

**********

3. Has the statistical analysis been performed appropriately and rigorously?

Reviewer #1: Yes

**********

4. Have the authors made all data underlying the findings in their manuscript fully available?

The PLOS Data policy

Reviewer #1: Yes

**********

5. Is the manuscript presented in an intelligible fashion and written in standard English?

Reviewer #1: Yes

**********

Reviewer #1: 1- Target selection rationale: Although LYN was selected for molecular dynamics simulations, Table 2 shows that MAPK14 has a substantially higher NetPredictor score. Please provide a clearer explanation for prioritizing LYN over MAPK14.

2- Docking validation: The manuscript states that the docking protocol was validated using RMSD; however, the actual RMSD values are not reported. Please include the validation results (e.g., RMSD values or a supplementary table) to support the reliability of the docking protocol.

**********

what does this mean?). If published, this will include your full peer review and any attached files.

If you choose “no”, your identity will remain anonymous but your review may still be made public.

**Do you want your identity to be public for this peer review?**  For information about this choice, including consent withdrawal, please see our Privacy Policy

Reviewer #1: No

**********

<!--a=1

# Editor report: Acceptance letter

**DOI:** 10.1371/journal.pone.0357093.r006

**Authors:** Eman Zahran

PONE-D-25-50743R2

PLOS One

Dear Dr. Ruengjitchatchawalya,

I'm pleased to inform you that your manuscript has been deemed suitable for publication in PLOS One. Congratulations! Your manuscript is now being handed over to our production team.

At this stage, our production department will prepare your paper for publication. This includes ensuring the following:

* All references, tables, and figures are properly cited

* All relevant supporting information is included in the manuscript submission,

* There are no issues that prevent the paper from being properly typeset

You will receive further instructions from the production team, including instructions on how to review your proof when it is ready. Please keep in mind that we are working through a large volume of accepted articles, so please give us a few days to review your paper and let you know the next and final steps.

Lastly, if your institution or institutions have a press office, please let them know about your upcoming paper now to help maximize its impact. If they'll be preparing press materials, please inform our press team within the next 48 hours. Your manuscript will remain under strict press embargo until 2 pm Eastern Time on the date of publication. For more information, please contact onepress@plos.org.

You will receive an invoice from PLOS for your publication fee after your manuscript has reached the completed accept phase. If you receive an email requesting payment before acceptance or for any other service, this may be a phishing scheme. Learn how to identify phishing emails and protect your accounts at https://explore.plos.org/phishing.

If we can help with anything else, please email us at customercare@plos.org.

Thank you for submitting your work to PLOS One and supporting open access.

Kind regards,

PLOS ONE Editorial Office Staff

on behalf of

Professor Eman Zahran

Academic Editor

PLOS One
